# Multiple-Race Stem Rust Resistance Loci Identified in Durum Wheat Using Genome-Wide Association Mapping

**DOI:** 10.3389/fpls.2020.598509

**Published:** 2020-12-17

**Authors:** Shitaye H. Megerssa, Karim Ammar, Maricelis Acevedo, Gina Brown-Guedira, Brian Ward, Ashenafi G. Degete, Mandeep S. Randhawa, Mark E. Sorrells

**Affiliations:** ^1^Plant Breeding and Genetics Section, School of Integrative Plant Science, Cornell University, Ithaca, NY, United States; ^2^International Maize and Wheat Improvement Center (CIMMYT), Mexico D.F., Mexico; ^3^Department of Global Development, Cornell University, Ithaca, NY, United States; ^4^USDA-ARS Plant Science Unit, Raleigh, NC, United States; ^5^Debre Zeit Agricultural Research Center, Ethiopian Institute of Agricultural Research (EIAR), Debre Zeit, Ethiopia; ^6^International Maize and Wheat Improvement Center (CIMMYT), Nairobi, Kenya

**Keywords:** durum wheat, genome-wide association, stem rust, multiple-race, major gene, field resistance

## Abstract

Stem rust of wheat caused by *Puccinia graminis* Pers. f.sp. *trtici* Eriks and E. Henn., is the most damaging fungal disease of both common (*Triticum aestivum* L.) and durum (*Triticum turgidum* L., ssp. Durum) wheat. Continuously emerging races virulent to many of the commercially deployed qualitative resistance genes have caused remarkable loss worldwide and threaten global wheat production. The objectives of this study were to evaluate the response of a panel of 283 durum wheat lines assembled by the International Maize and Wheat Improvement Center (CIMMYT) to multiple races of stem rust in East Africa at the adult plant stage and map loci associated with field resistance. The lines were evaluated in Debre Zeit, Ethiopia and Njoro, Kenya from 2018 to 2019 in five environments (year × season). The panel was genotyped using genotyping-by-sequencing. After filtering, 26,439 Single Nucleotide Polymorphism (SNP) markers and 280 lines and three checks were retained for analysis. Population structure was assessed using principal component analysis. Genome-wide association analysis (GWAS) was conducted using Genomic Association and Prediction Integrated Tool (GAPIT). The broad-sense heritability of the phenotype data revealed that 64–83% of the variation in stem rust response explained by the genotypes and lines with multiple race resistance were identified. GWAS analysis detected a total of 160 significant marker trait associations representing 42 quantitative trait loci. Of those, 21 were potentially novel and 21 were mapped to the same regions as previously reported loci. Known stem rust resistance genes/alleles were postulated including *Sr8a*, *Sr8155B1, SrWeb/Sr9h, Sr11, Sr12, Sr13/Sr13 alleles, Sr17, Sr28/Sr16*, *Sr22*, and *Sr49.* Lines resistant to multiple races in East Africa can be utilized as parents in durum wheat breeding programs. Further studies are needed to determine if there are new alleles at the *Sr13* locus and potential markers for the known *Sr13* alleles.

## Introduction

Durum wheat (*Triticum turgidum* L., ssp. Durum (Desf.) Husnot, 2n = 4× = 28; AABB genome) is among the tetraploid wheat species used for making pasta, couscous and other traditional recipes mainly consumed in the Mediterranean regions (Shewry and Hey, 2015). The European Union, Canada, the Mediterranean basins, the North American plains and Mexico are the major producers of durum wheat in the world (Bond and Liefert, 2017). A number of biotic and abiotic stress factors challenges the production of durum wheat. Among the biotic factors, stem rust of wheat caused by *Puccinia graminis* f.sp. *tritici* Eriks. & E. Henn (*Pgt*) is the most destructive fungal disease of both common and durum wheat (Roelfs et al., 1992). Stem rust can occur in all wheat growing areas and can cause complete yield loss under severe epidemics when susceptible cultivars are grown (Dean et al., 2012). The shriveling of grain due to stem rust can also downgrade the quality of the harvest and resulting end use products.

East African highlands are considered as hot spots for the emergence of new stem rust pathogen races. The emergence of new virulent races in East Africa and other parts of the world caused severe losses and continue to pose a threat to global wheat production and food security (Olivera et al., 2015; Singh et al., 2015; Bhavani et al., 2019). Many of the races evolve with corresponding virulence to commercially deployed resistance genes and some have broad virulence spectrum. The races in East Africa including Ug99 (TTKSK) and its lineage, TKTTF (“Digalu”), TRTTF and JRCQC defeated the resistance conferred by many major/R-genes in breeding lines and commercial cultivars. Stem rust race Ug99 was identified in Uganda in 1999 and spread across other countries in East Africa, the Middle East and South Africa. To date, 13 races identified from different countries with broad virulence to commercially deployed resistance genes, are considered part of the of the Ug99 lineage (Singh et al., 2015; Nirmala et al., 2017; Bhavani et al., 2019). Due to the continuously evolving races in the Ug99 group, most of the worldwide wheat germplasm were found to be moderately to highly susceptible to this group of races (Bajgain et al., 2015b; Singh et al., 2015).

Breeders in different regions of the world are incorporating resistance genes effective against the Ug99 lineages in their germplasm. However, the continuously emerging virulent races unrelated to Ug99 such as TKTTF, TRTTF, and JRCQC in East Africa (Olivera et al., 2015) and the rest of the world, continue to defeat major resistance genes effective against the Ug99 race groups, threatening global production of both common and durum wheat. Race TKTTF identified in Ethiopia during the 2013/14 epidemics caused close to 100% yield loss on 10,000 hectares of land planted with the wheat variety “Digalu.” This race defeated the resistance conferred by *SrTmp* which was effective against the Ug99 lineages. TKTTF has broad virulence to several other major genes (Olivera et al., 2015). Races JRCQC and TRTTF have combined virulence to the most frequent resistance genes/alleles in durum wheat, namely *Sr13b* and *Sr9e* that are effective against TTKSK and other races from the same lineage (Olivera et al., 2012). Due to the emergence of JRCQC, a very large proportion of the global durum wheat germplasm including many of the CIMMYT and North American durum wheat germplasm which were protected by *Sr9e* and *Sr13b* became susceptible in Ethiopia where this race is predominant. These two races also have broad virulence to other major *Sr* genes deployed in commercial cultivars. TRTTF is virulent to *SrTmp* and *Sr36* and was the first to defeat the resistance conferred by the 1AL-1RS rye translocation (*Sr1RS*) (Olivera et al., 2012). As a result all spring and winter wheat varieties carrying these genes became susceptible to *Pgt* races identified in Africa and Asia (Olivera et al., 2012; Singh et al., 2015). Among the alleles of *Sr13, Sr13a* is effective against races TTKSK, TKTTF, TRTTF, JRCQC and the race recently identified in Italy and Georgia (TTRTF) while *Sr13b* is effective only against TTKSK and TKTTF (Zhang et al., 2017; Olivera et al., 2019). These resistance alleles, unless deployed properly in combination with other genes, are likely to be defeated by an emerging race.

More than 60 stem rust resistance genes have been cataloged and about 34 of them are located in the A and B sub-genomes. However, most of them are R-gene/major-gene resistances and many are effective against specific races only (McIntosh et al., 1995, 2017). Among the cataloged *Sr* genes, only five confer adult plant resistance (APR), namely *Sr2, Sr55* (*Lr67/Yr46/Pm39*), *Sr56, Sr57 (Lr34/Yr18/Pm38)*, and *Sr58 (Lr46/Yr29/Pm39)* (Singh et al., 2015). Adult plant resistance (APR) is quantitative in nature controlled by several genes each with small effects and is thought to be more durable than the qualitative major gene-based resistance. Quantitative resistance is generally expressed at the adult plant stage and identified through field evaluations of seedling susceptible lines (Laidò et al., 2015). Conversely, evaluation of lines for field response regardless of their seedling response can be applied to identify all stage resistance genes but, selection for APR could be challenging due to the masking by major or R-genes. Deploying combinations of several APR genes or in combination with effective major genes is a possible strategy to increase the durability of resistance in stem rust management (Bhavani et al., 2011). The genetic characterization and identification of available sources of resistance in a given germplasm pool is important for the judicious use of different resistance sources and subsequent deployment of gene combinations with proper stewardship. Genetic studies characterizing sources of resistance to stem rust are more limited in durum wheat than in common wheat (Chao et al., 2017). The limited genetic studies in the past used low density markers such as simple sequence repeats (SSRs) and Diversity arrays technology (DArT) (Haile et al., 2012; Letta et al., 2013) and very few used high density SNP markers. The lines used in the current study were not previously characterized for their field responses to the multiple stem rust races currently prevailing in East Africa and their genetic basis of resistance was not well-understood. In the current study, a panel of lines from the CIMMYT germplasm pool were evaluated against multiple races of stem rust in Ethiopia and Kenya, and we used high density SNP markers discovered through the Genotyping-by-sequencing (GBS) approach to identify genomic regions associated with the field responses of the genotypes.

## Materials and Methods

### Plant Materials and Phenotyping

A panel of 283 spring durum wheat genotypes composed of a wide collection of advanced breeding lines and some cultivars that represent the current CIMMYT durum wheat germplasm was evaluated for adult plant response to stem rust for three seasons in Ethiopia (Debre Zeit Agricultural Research Center); off-season (January to May) 2018 and 2019, main season (June to November) 2018; and two seasons in Kenya (KARI, Njoro Station) during the main season (June to October) 2018 and 2019; hereafter abbreviated as ETOS18, ETOS19, ETMS18, KNMS18, and KNMS19, respectively. Among the 283 genotypes included in the panel, 10 harbor *Sr25* (translocation from *Thinopyrum ponticum* onto chromosome 7A), 6 carry the *Sr25*+ *Sr22 (Sr22* is a translocation from *T. boeticum* onto chromosome 7A), and 8 have *Sr38* (a translocation from *T. ventricosum* onto chromosome 2A) that were developed through marker-assisted selection and represent resistances that are not present in any of the durum germplasm pools worldwide (Ammar, personal communication, 2020). In the Debre Zeit nursery, lines were planted in dual rows of 1 m length with 0.2 m inter-row spacing arranged in randomized incomplete block design with two replications. Two susceptible (“Arendato” and “Local red”) and one moderately resistant (“Mangudo”) checks were repeated after every 50 plots. In addition, the 20 stem rust differential lines with known stem rust resistance genes (Fetch et al., 2009) were planted at the beginning and end of the nursery in Debre Zeit, Ethiopia. The plots were surrounded by spreader rows planted with a mixture of susceptible lines, namely “Arendato,” “PBW 343,” “Morocco,” and “Digalu” in equal proportions. In the Njoro nursery, plots consisted of two rows of 0.7 m with 0.3 m inter-row spacing arranged using the same design as in Ethiopia. The plots and the experimental field were surrounded by spreader rows planted as hill plots with an equal proportion mixture of the stem rust susceptible cultivars “Cacuke” and “Robin,” and six lines carrying *Sr24* (Genotype identification number (GID) = 5391050, 5391052, 5391056, 5391057, 6391059, and 5391061).

Disease infection was initiated by artificial inoculation of the spreader rows with a bulk of stem rust urediniospores collected at each specific location from the previous season to ensure uniform disease distribution in the trials. Spreaders were inoculated with a mixture of field collection of stem rust races TTKSK, TKTTF, JRCQC, TTTTF, and TRTTF in Debre Zeit, Ethiopia; and races TTKSK, TTKST, TTKTT, and TTTTF in Njoro, Kenya. Inoculation was done by suspension of urediniospores in distilled water and adding a drop of Tween 20 (a drop/0.5 lt) and syringe-injection of the spreader rows (at ∼30 cm interval per meter) at stem elongation (∼Zadok’s growth scale 31, first node detectable) (Zadoks et al., 1974) and repeated two to three times. Then urediniospores prepared with a similar protocol were sprayed one to two times on the spreader rows to enhance infection and disease development. In the off-season nurseries, furrow irrigation was applied for the establishment of the nursery and for providing a humid environment for proper disease development.

Disease severity was scored according to the modified Cobb’s scale by estimating the proportion of the stem area (0–100%) covered by rust pustules (Peterson et al., 1948). Infection response was scored according to Roelfs et al. (1992) based on the size of pustules and amount of chlorosis and necrosis on the stem. The responses classes are: “0” for no visible infection, “R” for resistant, “MR” for moderately resistant, “MS” for moderately susceptible and “S” for susceptible. A combination of responses was scored in the case of an overlap of infection responses on a single genotype by taking the most frequent response first followed by the less frequent. Stem rust was scored two to four times in each environment at 8–10-day intervals and the final scoring was considered for analysis. The stem rust severity and response were combined in a value called coefficient of infection (CI) calculated by multiplying the severity values with a linearized scale of 0–1 assigned to the respective responses. The scale was assigned as: immune = 0.0, *R* = 0.2, *MR* = 0.4, *MS* = 0.8 and *S* = 1.0, and the mean of the scale of responses was used to calculate CI in the cases where combinations of infection responses were scored for a given genotype (Stubbs et al., 1986).

### Statistical Analysis of Phenotype Data

The CI was used in the statistical analysis using R statistical software version 3.6.1 (R Core Team, 2019) and ASReml-R version 3 for spatial correction (Gilmour et al., 2009). We fitted different models and finally chose a model which resulted in the highest estimate of broad-sense heritability. In some cases, a model with a significant Wald test for fixed effect was considered when the row and column effects were fitted as fixed (Gilmour et al., 2009). For the off-season 2018 nursery in Ethiopia, a linear mixed model (LMM) described in Eq. 1 was fitted on the CI using ASReml-R to extract the best linear unbiased predictions (BLUPs).

(1) y=ijkμ+g+iC+jr+kεijk

Where: y_*ijk*_ is the response of the *i*th line in the *j*th column and the *k*th replication, g_*i*_ is the random effect of the *i*th line, C_*j*_ is the fixed effect of the *j*th column, and *r*_*k*_ is the random effect of *k*th replication and ε_*ijk*_ is the residual associated with the model.

For the main season 2018 nursery in Ethiopia, the LMM described in Eq. 2 was fitted on the square-root-transformed CI using the lmer() function of the R package lme4 (Bates et al., 2015) and extracted genotypic BLUPs (R Core Team, 2019).

(2) $$y_{ij}=\mu +g_{i}+r_{j}+e_{ij}$$

Where: y_*ij*_ is the response of the *i*th line at the *j*th replication, g_*i*_ is the random effect of the *i*th genotype (line), r_*j*_ is the random effect of the *j*th replication, ε_*ij*_ the residual associated with the model.

For the off-season 2019 nursery in Ethiopia, the LMM described in Eq. 3 with the residual variance (ε_*ij*_) fitted as ar1(row):ar1(column), the first order autoregressive correlation of the residuals of the row and column, as random effects, which assumes the residuals could be correlated (Gilmour et al., 2009) was fitted on the square-root transformed CI using ASReml-R and BLUPs were extracted. For the nursery in Kenya during the main season 2018, the LMM described in Eq. 3 was fitted on the square-root-transformed CI using ASRreml-R (Gilmour et al., 2009)) and genotypic BLUPs were extracted.

(3) $$y_{ijkl}=\mu +g_{i}+R_{j}+C_{k}+r_{l}+\varepsilon_{ijkl}$$

Where: y_*ijkl*_ is the response of the *i*th line in the *j*th row, in the *k*th column and *l*th replication, g_*i*_ is the random effect of the *i*th line, *R*_*j*_ the fixed effect of the *j*th row, *C*_*k*_ is the fixed effect of the *k*th column, r_*l*_ is the random effect of the *l*th replication and ε_*ijkl*_ is the residual associated with the model.

For the main season 2019 nursery in Kenya, the MLM described in Eq. 2 was fitted on the square-root transformed CI using the lmer() function of the R package lme4 and genotypic BLUPs were extracted. From the variance components estimated from each model, broad sense heritability was calculated following the method by Holland et al. (2003).

(4) H=2V/gVp

Where: *H*^2^ is the broad sense heritability, *V*_*g*_ is the variance due to the genotype (line), *V*_*p*_ is the variance due to the phenotype, *V*_*p*_ = *V*_*g*_ + *V*_*e*_, *V*_*e*_ is the residual variance.

### Genotyping and Data Filtering

Two cm-long young leaf tissues were collected and frozen at −80^o^C for 2 weeks. The frozen leaf samples were then lyophilized and shipped to the USDA-ARS Eastern Regional Small Grains Genotyping Laboratory in Raleigh, NC for genotyping. Genomic DNA was isolated from the lyophilized tissue samples using a sbeadex plant DNA isolation kit (LGC Genomics, Middlesex, United Kingdom) according to manufacturer’s instructions. Genomic DNA was then fragmented using a *PstI-MspI* double restriction digest following the GBS protocol of Poland et al. (2012). Sequencing adapters were ligated to DNA fragments, and single-ended 100 bp short read sequencing was then performed on an Illumina (San Diego, CA) Novaseq instrument. SNP genotype calling was done using TASSEL software version 5 (Glaubitz et al., 2014) and the recently published durum wheat reference genome of cultivar “Svevo” (Maccaferri et al., 2019) was used to assign a physical position to each SNP marker. Thereafter, SNP markers with missing data above 50%, minor allele frequency (MAF) below 5%, and heterozygous call rates above 15% were filtered out. Missing data was then imputed using Beagle 5 (Browning et al., 2018). Following imputation, PLINK 1.9 (Chang et al., 2015) was used to remove all but one SNP in groups of SNPs in perfect linkage disequilibrium (LD) with each other (*r*^2^ = 1), using a sliding window of 250 SNPs, advancing by 10 SNPs per step. In total, 26,439 SNPs were called in 283 lines (including three checks) and retained for genome-wide association analysis.

All lines were also screened with kompetitive allele-specific PCR (KASP) assays developed around SNP linked to the resistance genes *Sr2* and *Lr46/Sr58*. For *Sr2*, lines were evaluated with marker *Sr2_ger93p* (Mago et al., 2011). For *Sr58*, lines were evaluated for SNP CIMwMAS0085 tightly linked leaf rust APR gene, *Lr46*^1^. Lines were also evaluated with a KASP assay targeting *Sr13*, the major gene most frequent in durum wheat which provides effective resistance to the Ug99 lineage. The *Sr13* assays was designed around the mutation at amino acid W743R (Zhang et al., 2017). Lines having the 734R amino acid associated with resistance to TTKSK were noted as having an *Sr13* allele for resistance. KASP assay primer sequences are noted in Supplementary Table 10.

### Population Structure and Linkage Disequilibrium Analyses

If not taken into account, population structure results in false positive marker trait associations (MTA) in GWAS analyses. In the current study, the presence of population structure was assessed using principal component analysis (PCA) using the R function “prcomp” and visualized for the clustering of PC scores. The extent of LD in a population is useful for determining the resolution of association mapping. The LD between pairs of markers for the 26,439 markers was calculated as the squared allele frequency correlation (r^2^) by applying a sliding window of 50 markers using TASSEL software version 5 (Bradbury et al., 2007). The r^2^ values of pairs of loci were plotted against the physical distances in Megabases (Mb) after randomly sampling 10% of the total loci pairs. A locally estimated scatterplot smoothing (LOESS) curve was fitted using “geom_smooth” in R package ggplot2 (Wickham, 2016) to visualize the decay of LD in each of the 14 chromosomes. The r2 threshold to verify that LD was likely to be due to linkage was estimated from the 95th percentile of the distribution of the square-root-transformed r^2^ of unlinked markers (Breseghello and Sorrells, 2006). The point at which the horizontal line at the r^2^ critical value and the LOESS curve on the LD scatter plot intersected was treated as the estimate of the extent of LD for each chromosome in our study population.

### Genome Wide Association Analysis

The BLUPs derived from the respective models fitted on the phenotypic data were considered as the response to fit GWAS models. The analysis was conducted using GAPIT by fitting four models; Mixed Linear Model (MLM) (Lipka et al., 2012), Compressed Mixed Linear Model (CMLM) (Zhang et al., 2010), Multi-locus Mixed Linear Model (MLMM) (Segura et al., 2012), and Fixed and random model Circulating Probability Unification (FarmCPU) (Liu et al., 2016). MLM is a single locus model that fits one marker at a time as a fixed effect, population structure as a fixed effect (Q) and marker based additive relationship matrix or Kinship (K) as a random effect in the model (Q+K model). CMLM fits MLM after clustering individuals to estimate kinship and reduces computational time (Zhang et al., 2010). MLMM estimates variance components using a stepwise forward-backward linear mixed-model regression and fits the significant SNP as a covariate for the following step (Lipka et al., 2012), and FarmCPU uses both Fixed Effect and Random Effect models iteratively. It fits one marker at a time in the Fixed Effect Model with significant markers as covariates. Then the kinship of the significant markers is used to fit the Random Effect Model (Liu et al., 2016). The first two PC scores were used to account for population structure in all models. A False Discovery Rate (FDR) of 5% was applied for multiple comparison adjustment and as a threshold to declare significant marker-trait associations (MTAs) (Benjamini and Hochberg, 1995). The deviation of the observed −log10 *p*-value distribution from the expected distribution in the quantile-quantile (Q-Q) plots was used to compare the models and results were interpreted from MLM and FarmCPU. Manhattan plots of −log10 *p*-values were generated using the R package qqman (Turner, 2017). A linkage disequilibrium heatmap was plotted for significant markers on chromosome 6A and the *Sr13* marker, and the significant markers on chromosome 7A using the R package LDheatmap applied on the square matrix of the squared allele frequency correlation between pairs of markers (Shin et al., 2006). Significant markers tagging quantitative trait loci/locus (QTL) were gathered from previous QTL studies on durum and common wheat. The sequences of these markers were searched from the GrainGenes database. Then the fasta file of the sequences was aligned against the respective chromosomes of the “Svevo” reference sequence using the blastn program of the IWGSC database for similarity of physical positions with the significant markers identified in the current study and for postulation of resistance genes/alleles.

## Results

### Phenotypic Data Analyses

**FIGURE 1:**
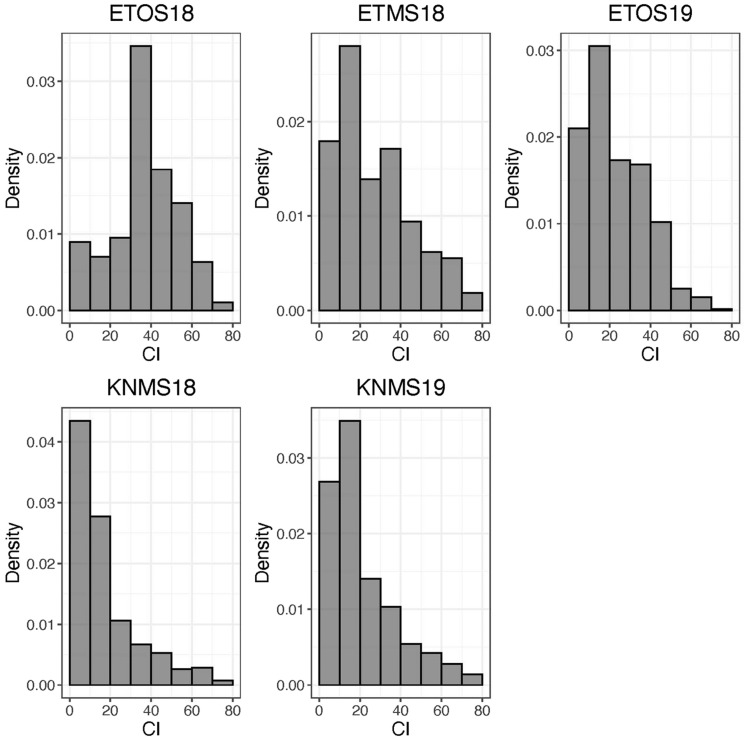
Distribution of coefficient of infection (CI) calculated as the product of severity and a linearized scale for response across five environments.

The distributions of the CI were skewed toward resistance in all environments except ETOS18 which was close to normal distribution (Figure 1). The percentage of resistant lines (*CI* ≤ 18) varied from 10% in ETOS18 with a mean CI of 40–65% in KNMS18 with a mean CI of 18.3 (Table 1). The broad-sense heritabilities estimated from the variance components of each model fitted were 0.71 for ETOS18, 0.64 for ETMS18, 0.83 for ETOS19, 0.77 for KNMS18 and 0.69 for KNMS19 indicating that most of the variation in the response (64–83%) was explained by the genotypic component.

**TABLE 1:** Summary of descriptive statistics, genetic variance and broad-sense heritability of coefficient of infection (CI) of the 283 durum wheat lines across the five environments.

Statistic	ETOS18	ETMS18	ETOS19	KNMS18	KNMS19
Mean	40.0	28.7	24.4	18.3	25.1
Range	0–80	0–80	0–80	0–90	1–100
Resistant (%)	10	35	46	65	55
Susceptible (%)	90	65	54	35	45
V_*g*_	241	2.58	2.36	3.44	3.39
H^2^	0.71	0.64	0.83	0.77	0.69

*H^2^ Broad-sense heritability. V_g_ genetic variance.*

Screening of the lines with markers linked to *Sr2*, *Sr13*, and *Sr58* (using *Lr46* linked marker) revealed that 69% of the total number of lines evaluated were likely to carry *Sr13*, 46% were likely to have *Lr46* (*Sr58*), 30% (85 lines) were likely to have both genes (*Sr13* and *Lr46/Sr58*) and 15% (43 lines) were lacking both genes. Among the lines positive to *Sr13* and *Lr46/Sr58*, 14.3% showed resistance (*CI* ≤ 18) in all the five environments, 16.7% in four environments, 32.1% in three environments 21.4% in two environments and 15.5% in a single environment (Supplementary Table 1). Three lines with an Origin GID 7147179, 7147180, 7147182 showed immune responses in most environments (Supplementary Table 1). None of the lines from the current panel was found to carry *Sr2*. Among the 43 lines that lack *Sr13* and *Lr46/Sr58* based on the marker screening, a line with GID 7145241 was consistently resistant in all the five testing environments, line GID 6951159 was resistant in four environments except ETOS19, line GID 5928165 was resistant in three environments, line GID 7408527 was resistant in ETOS19 and KNMS18, line GID 7409573 was resistant in KNMS18 and KNMS19. Lines with GID 7383430, 7407575, and 7384241 were resistant in KNMS18 while GID 7408885 was resistant in KNMS19 (data not shown).

### Population Structure and Linkage Disequilibrium Analyses

**FIGURE 2:**
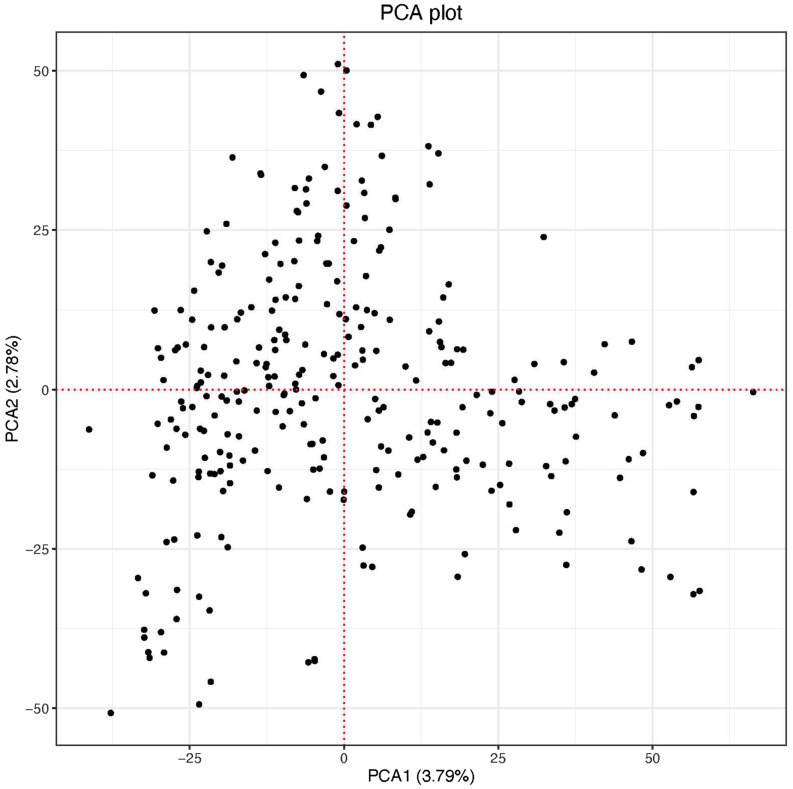
Principal component-1 (PC1) plotted against principal component-2 (PC2) of the panel.

The scatter plot of the first two PC scores indicated two putative groups although the clustering was not no clear. The first and the second PC scores explained 3.79 and 2.78% of the genetic variation in the panel, respectively (Figure 2). The genome-wide LD calculated for the 26,439 markers resulted in a total of 1,320,675 pairwise comparisons of loci. Out of the total pairs of loci compared, 37.4% (494,449) were in significant LD (*p <* 0.001). The mean genome-wide LD (r^2^) for the population was 0.39. Of the total loci pairs, 1.28% (16,860) of the loci pairs were in wide range LD on different chromosomes, and 1.09% (184) of those on different chromosomes were in significant LD (*p* < 0.001). The LD threshold for the population estimated from the 95th percentile of the distribution of square root transformed r^2^ of unlinked markers (markers located on different chromosomes) was 0.16, the critical value beyond which LD was likely due to physical linkage. The decay of LD for the linked markers varied across chromosomes in both sub-genomes (Supplementary Figure 1). The LOESS curve crossed the horizontal line of threshold value at approximately 4 Mb in all chromosomes of the A genome except chromosomes 2A (8 Mb), 3A (3 Mb), and 5A (5 Mb) with an average of 4.5 Mb. For the B genome, the LOESS curve crossed with the horizontal line of the critical value at 5 Mb for chromosomes 1B, 2B, and 7B, at 4 Mb for chromosomes 3B and 5B, at 8 Mb for 4B, and at 4.5 Mb for 6B with an average of 4.6 Mb. The decay of LD in chromosome 2A and 4B was slower (8 Mb) than the rest of the chromosomes (Supplementary Figure 1).

### Genome-Wide Association Analyses

GWAS analysis was conducted by fitting four models (MLM, CMLM, MLMM, and FarmCPU) for each of the evaluation environments. Based on the Q-Q plots and the power of FarmCPU to limit potential false positive and false negative associations, we limited the interpretation of results to those from MLM and FarmCPU models. Many of the significant MTAs identified by MLM were confirmed by FarmCPU and the unconfirmed MTAs were assessed for consistency across environments to determine if they were reliable MTAs (Supplementary Tables 2–7). FarmCPU selected the most significant marker from linked markers falling within the same QTL, such as for chromosomes 6A and 7A in the GWAS results of the MLM. FarmCPU also identified novel as well as previously reported MTAs unidentified by MLM (Supplementary Table 2). The results of the CMLM and MLMM were not considered further for the following reasons: the Q-Q plot of CMLM fitted the data well only for ETOS18, ETOS19, and KNMS18 and under such circumstances, the significant MTAs identified by MLM and CMLM were the same. Although MLMM had an acceptable Q-Q plot, this model identified the fewest significant MTAs in all the five environments (data not shown).

MLM identified a total of 135 significant MTAs for field resistance to multiple *Pgt* races in Ethiopia and Kenya across the five testing environments. From these 14.1% were detected in all the five environments, 7.4% in four environments, 5.2% in three environments, 16.3% in two environments and 57% in only one environment (Supplementary Tables 3–8). Among the 57% (77 markers) identified in a single testing environment, most were on chromosomes 6A and 7A and they were in LD with other nearby markers identified across multiple environments (Figures 5, 6). From the total MTAs identified by MLM, 9.6% were confirmed by FarmCPU (Supplementary Tables 2, 7) and most of the significant markers on chromosome 6A and 7A identified by MLM were in LD with the those identified by FarmCPU on the same chromosome. FarmCPU identified a total of 47 significant MTAs (Supplementary Table 2). Among the total, 4% were identified in three testing environments, 11% in two environments and the remaining 85% in a single testing environment (Table 2). Out of the total MTAs identified by the two models, nine MTAs were on unaligned contigs (Supplementary Tables 2–7).

**TABLE 2:** Lists of consistent significant markers between environments identified using FarmCPU.

Position	Chr.	MAF	Environment	Proposed gene
724,805,496	3B	0.104	ETOS18, KNMS18	*Sr12*
691,693,264	5B	0.051	ETOS18, ETMS18	*Sr49*
692,277,095	5B	0.058	ETOS19, KNMS18	*Sr49*
592006	6A	0.228	ETOS18, KNMS19	*Novel/Sr8155B1*
612,043,936	6A	0.302	ETMS18, KNMS18, KNMS19	*Sr13*
700,805,183	7A	0.076	ETOS18, ETOS19, KNMS19	Reported
717,518,884	7A	0.058	ETMS18, KNMS18	Reported

**FIGURE 3:**
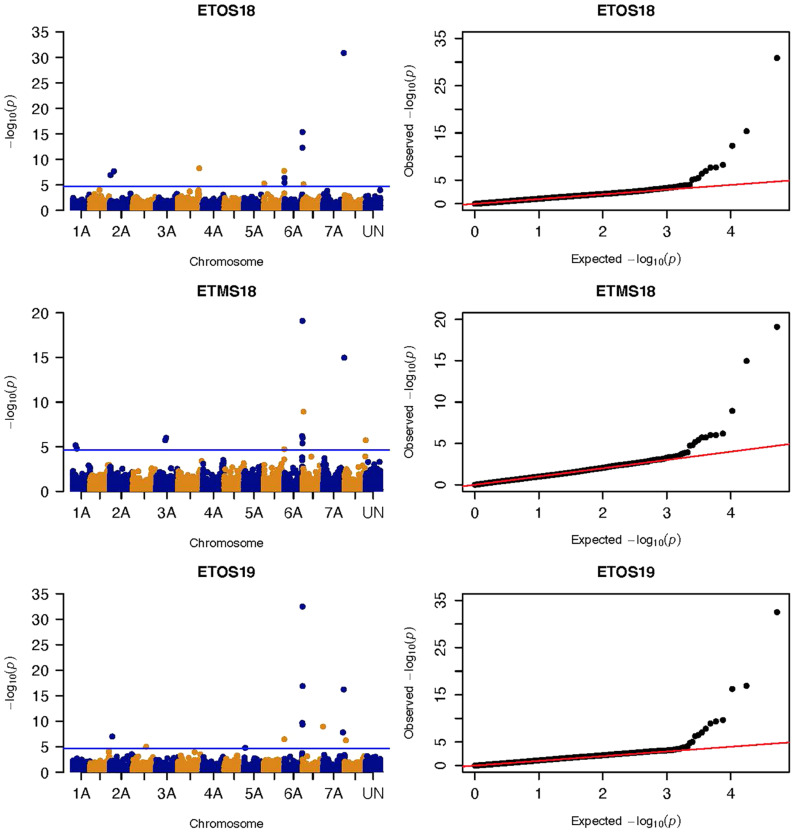
Manhattan and QQ-plots of GWAS results of field resistance of durum wheat lines to multiple races in Ethiopia across three seasons identified using FarmCPU.

**FIGURE 4:**
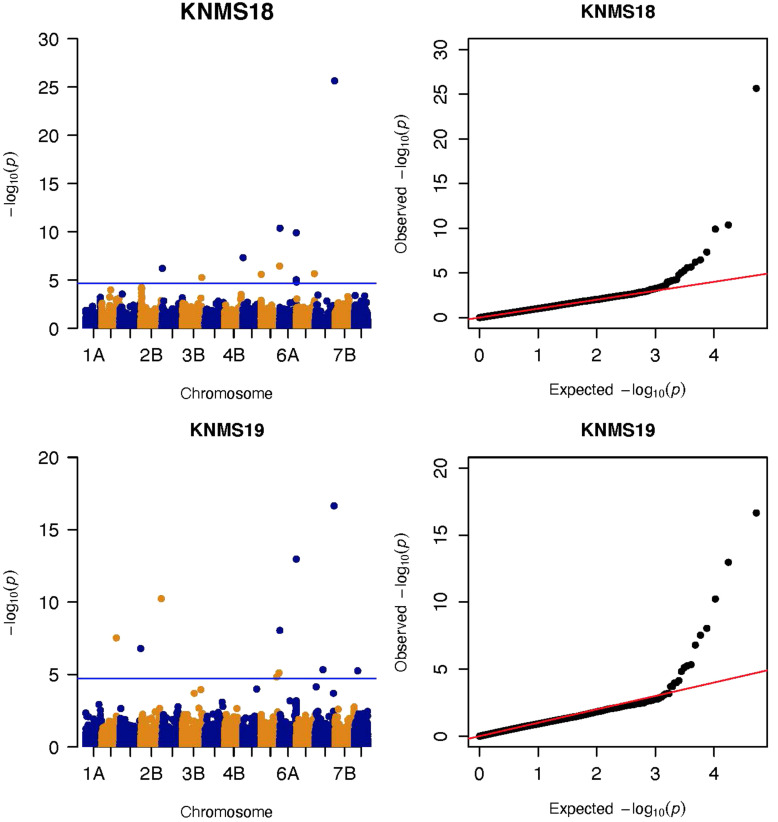
Manhattan and QQ-plots of GWAS results of field resistance of durum wheat lines to multiple races in Kenya across two seasons identified using FarmCPU.

Three significant MTAs were identified on chromosome 1A at 95, 144, and 485 Mb (Figure 3 and Supplementary Figure 2). The QTL at 95 and 485 Mb explained 3 and 3.73% of the phenotypic variation, respectively, and the MTA at 144 Mb was close to the threshold (FDR adjusted *p* = 0.04) (Supplementary Tables 2, 5). On chromosome 1B, four significant MTAs were identified at 183, 546, 587, and 620 Mb (Supplementary Figure 2 and Figure 4). The three MTAs on chromosome 1B except the 183 Mb (FDR adjusted *p* = 0.045) represented three QTL that explained 3.43–4.59% of the phenotypic variation (Supplementary Tables 2–4). Seven significant MTAs (20, 67, 78, 135, 699, 728, and 770 Mb) were detected on chromosome 2A (Figures 3, 4). Six of the MTAs represented putatively six QTL and one at 699 Mb had an FDR adjusted *p*-value close to the threshold (0.049) (Supplementary Table 3). Four MTAs (56, 456, 759, and 780 Mb) were identified on chromosome 2B (Supplementary Figure 2 and Figures 3, 4). The three MTAs represented three QTL that explained 2.37–3.93% of the phenotypic variation while the 56 Mb region was close to the threshold (FDR adjusted *p* = 0.046) (Supplementary Table 3). Three putative QTL represented by three significant MTAs (9, 313, and 344 Mb) were identified on chromosome 3A using FarmCPU (Figures 3, 4). The phenotypic variance explained by the two MTAs at 313 and 344 Mb was 3.25 and 2.98%, respectively, and was very low for the 9 Mb region (data not shown). All the significant MTAs identified on chromosomes 1A, 1B, 2A, and 2B were identified at a single testing environment and using either one of the two models.

Five significant MTAs (38, 55, 97, 213, and 724 Mb) representing three QTL were detected on chromosome 3B. The MTA at 55 Mb was identified at a single environment using MLM and it explained 4.04% of the phenotypic variation. The 97 Mb region identified using MLM was consistent across four (ETOS18, ETMS18, ETOS19, KNMS18) of the five testing environments and it explained 3.91–4.81% of the phenotypic variation (Supplementary Tables 2–6). The QTL at 724 Mb was consistent across two testing environments (ETOS18 and KNMS18) and the two models (Table 2). This QTL (724 Mb) explained 3.28% of the phenotypic variation on average (Supplementary Table 3). The two MTAs at 38 and 213 Mb were close to the FDR threshold (FDR adjusted *p* = 0.04) (Supplementary Table 3). Two significant MTAs representing two putative QTL were identified on chromosome 4A using MLM. The 619 Mb region was consistent in all the five testing environments and explained 5–7.84% of the phenotypic variation while the association at 651 Mb region was detected in a single environment and explained 3.99% of the phenotypic variation (Supplementary Table 3–7). Two significant MTAs (8 Mb and 35 Mb) representing two putative QTL were detected on chromosome 5A using FarmCPU. These two MTAs were identified in one testing environment (Supplementary Table 2) and explained only 2.66 and 1.71% of the phenotypic variation, respectively (data not shown). Seven MTAs (at 12, 13, 581, 671, 688, 691, and 692 Mb) representing five QTL were identified on chromosome 5B (Figures 3, 4 and Supplementary Figures 2, 3). The QTL represented by the MTAs at 12 Mb and 13 Mb (LD, *r*^2^ = 0.46) was identified using FarmCPU in KNMS18 and ETOS18, respectively (Supplementary Table 2). This QTL explained 2.6% of the phenotypic variation on average (data not shown). The QTL at 581 Mb was consistently identified by MLM and FarmCPU in KNMS19 and explained 5.56% of the phenotypic variation. Two QTL represented by single markers at 671 Mb and 688 Mb regions explained 3.17 and 3.63% of the phenotypic variation, respectively, and both were identified in one testing environment and one of the two models (Supplementary Tables 2, 3). The QTL at 691 Mb and 692 Mb identified by FarmCPU (LD, *r*^2^ = 0.86) was consistent across four of the five testing environments (Table 2).

**FIGURE 5:**
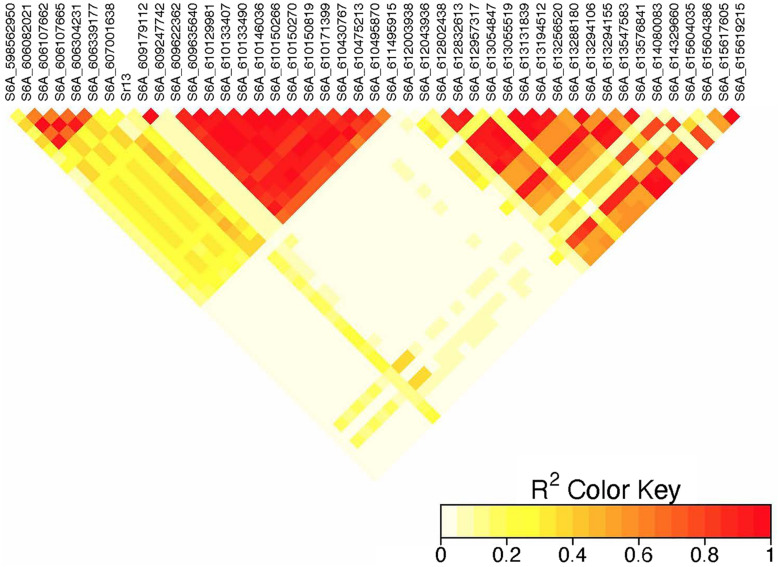
Linkage disequilibrium heatmap of the *Sr13* marker and nearby significant markers on chromosome 6A.

On chromosome 6A, 52 significant MTAs representing five putative QTL were identified using MLM and FarmCPU (Supplementary Tables 2–8). The MTA at 592 kb identified using FarmCPU was consistent across two environments (Table 2) and explained 2.68% of the phenotypic variation on average (data not shown). This marker (592,006 bp) was in strong LD (*r*^2^ = 0.89) with a significant marker at 4 Mb (4,914,394 bp) identified using FarmCPU which explained 3.18% of the phenotypic variation. An MTA identified by FarmCPU in a single environment at 1.4 Mb explained 3.18% of the phenotypic variation (data not shown). A QTL at 28 Mb was consistently identified at two testing environments and explained 4.42% of the phenotypic variation on average while the 334 Mb region was consistent across all the five testing environments and explained 3.52–7.39% of the phenotypic variation (Supplementary Table 4). Forty-five MTAs extending from 606 to 615 Mb represented one putative QTL on chromosome 6A that explained 3.38–9.79% of the phenotypic variation. All the significant markers identified on chromosome 6A that extended from 598 to 615 Mb except one marker at 612 Mb were in LD with the *Sr13* marker (*r*^2^ = 0.10–0.40) (Figure 5). The 598 Mb region was identified in a single environment and contributed less to the variation in the phenotype (*R*^2^ = 1.62%). Twenty-three MTAs identified by MLM extending from 609 Mb to 615 Mb were consistent across two to four testing environments (Supplementary Table 4), whereas nine MTAs from 606 to 615 Mb were consistently identified by MLM and FarmCPU (Supplementary Tables 2–7). One MTA at 612 Mb was consistently identified across three testing environments using FarmCPU (Table 2). From the MTAs on chromosome 6A that extended from 606 to 615 Mb, the most significant markers were located at 612 Mb (612,802,438 bp) (*p* = 1.01E-07) for ETOS18, at 611 Mb (611,495,915 bp) for ETMS18 (*p* = 8.47E-07) and ETOS19 (*p* = 5.61E-10), at 612 Mb (612,043,936 bp) for KNMS18 (*p* = 3.13E-09), and KNMS19 (*p* = 3.71E-09). The marker at 611 Mb (611,495,915 bp) was consistent across two testing environments and the two models. This MTA explained 5.31–9.49% of the phenotypic variation and this marker was in weak to strong LD (*r*^2^ = 0.12–0.75) with 22 significant markers that extended from 598 Mb to 610 Mb (Figure 5). The MTA at 612 Mb (612,043,936 bp) was consistently identified across four environments using MLM and three testing environments using FarmCPU (Supplementary Table 4 and Table 2). This MTA explained 3.44–9.79% of the phenotypic variation across the test environments. The other most significant marker at 612 Mb (612,802,438 bp) was consistent across three environments and the two models; it explained 4.94–9.29% of the phenotypic variation. This marker was in weak to strong LD (*r*^2^ = 0.14–0.96) with 20 significant markers that extended from 612 to 615 Mb on chromosome 6A (Figure 5).

Six significant MTAs were detected on chromosome 6B (Figures 3, 4 and Supplementary Figure 2). A QTL at 30 Mb and 31 Mb (LD, *r*^2^ = 0.33) identified using FarmCPU was consistent across two seasons in Ethiopia (Table 2) and explained only 2.36% of the phenotypic variation on average (data not shown). The MTAs at 666 and 692 Mb were identified in single environments using FarmCPU (Supplementary Table 2). The QTL at 666 Mb explained 2.35% of the phenotypic variation while the 692 Mb region contributed very low to the phenotypic variation (data not shown) and had low MAF (0.053). A QTL at 686 Mb and 687 Mb (LD, *r*^2^ = 0.64) was identified using MLM in ETOS19 and explained 3.72% of the phenotypic variation on average (Supplementary Table 5).

**FIGURE 6:**
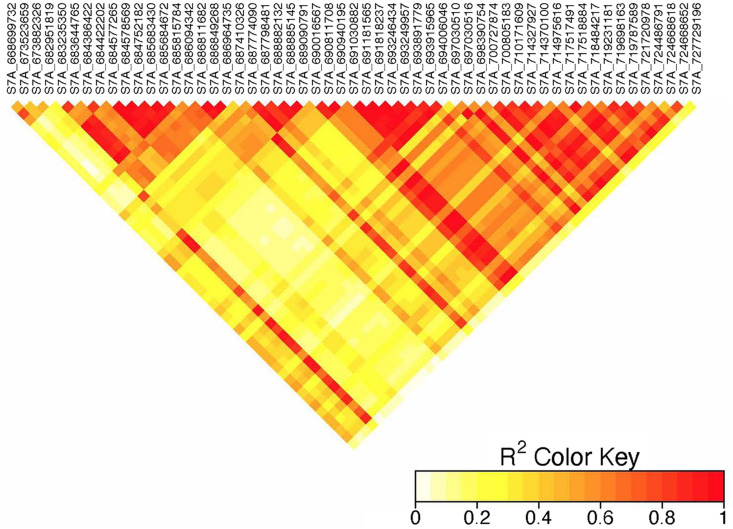
Linkage disequilibrium heatmap of adjacent significant markers on chromosome 7A.

On chromosome 7A, 60 significant MTAs were identified using MLM and FarmCPU (Figures 3, 4 and Supplementary Figures 2, 3). Four MTAs at 43, 117, 139, and 285 Mb regions were inconsistent across the testing environments and the two models. The remaining MTAs that extended from 668 to 727 Mb (55 Markers) explained 3.42–10.38% of the phenotypic variation (Supplementary Tables 3–7). These markers were in weak to strong LD and may represent the same QTL (Figure 6). On chromosome 7A, 23 MTAs that extended from 690 to 724 Mb identified using MLM were consistent across two to five testing environments (Supplementary Table 4). Two MTAs (700 and 717 Mb) were consistently identified by MLM and FarmCPU in all the five testing environments (Table 2). The markers at 700 Mb (700,805,183 bp) and 717 Mb (717,518,884 bp) were identified as the most significant markers in each of the testing environments using MLM and FarmCPU (Supplementary Tables 2–7). The MTA at 700 Mb explained 5.25–9.05% the phenotypic variation across the five testing environments (average = 7.13%) while the one at 717 Mb explained 5.06–10.38% of the phenotypic variation across the five testing environments (average = 7.66%). These two markers (700 and 717 Mb) were in strong LD (*r*^2^ = 0.83) (Figure 6). Five MTAs representing four QTL were identified on chromosome 7B. Two QTL at 46 and 717 Mb detected by FarmCPU and one QTL at 707 Mb detected by MLM were identified in single environments. A QTL at 622 and 644 Mb (LD, *r*^2^ = 0.64) identified by MLM was consistent across four of the five environments and explained 3.78–5.77% of the phenotypic variation (Supplementary Tables 2–7).

## Discussion

The characterization and identification of widely effective resistance available in breeding program’s elite pool is valuable for addressing the stem rust problem in durum wheat. In the current study, we evaluated the reaction of a panel of 283 elite durum wheat lines and cultivars representing the CIMMYT germplasm pool to multiple races of stem rust in East Africa and mapped a number of previously reported and novel genomic regions associated with field resistance to the locally prevailing races (Lists of Pedigrees: Supplementary Table 9).

### Phenotypic Data Analysis

The skewed distribution of the lines toward the resistance side in all testing environments except in ETOS18 could be due to the differences in race compositions across the testing environments (Figure 1). In contrast to races in Kenya which are less virulent on durum wheat, those in Ethiopia are composed of races such as the JRCQC with combined virulence to the most deployed stem rust resistance genes/alleles (*Sr13b* and *Sr9e*) in worldwide durum wheat germplasm and cultivars (Olivera et al., 2012). The similar frequency distribution of the CI of the lines in ETMS18 and ETOS19 to that of the two seasons in Kenya is not expected (Figure 1). The possible explanation for this result is that the spores collected in the previous season to inoculate the ETMS18 and ETOS19 trials are possibly composed of high frequency of durum avirulent races than virulent ones. Among the resistant lines across the five testing environments, 85 lines were likely carrying *Sr13* and *Lr46* which showed resistance against multiple stem rust races in single testing environment (15.5%) and all the five testing environments (14.3%) (Supplementary Table 1). This inconsistency in the response across environments while carrying these two genes could be due to the seasonal variation in race composition, race specificity of R-genes/alleles such as the alleles of *Sr13* since the marker used for screening of the lines for this gene was not allele specific and the subjectivity of disease scoring may also contribute. Lines lacking *Sr13* and *Lr46* that showed resistance to multiple-races across the testing environments may carry other resistance genes. These lines harboring widely effective field resistance would represent potentially useful parents that can be utilized in durum wheat breeding programs. Moreover, the risk of introducing linked undesirable alleles in utilizing these lines as sources of resistance in durum wheat breeding programs is unlikely since the study population is a collection of breeding lines from the CIMMYT durum wheat breeding program. Evaluating the multiple race resistant lines for agronomic performance and combining more resistance genes/alleles to the best performing lines can increase durability of resistance to stem rust in future varieties.

### Population Structure and Linkage Disequilibrium

The population structure in the current study panel was minimal indicated in the PCA plot and the variance explained by the two PCs (Figure 2). This could be because our study population was a panel of breeding lines sourced only from CIMMYT. The resolution of GWAS mapping relies on the level of LD, which can vary based on the population used for study (Chao et al., 2017). For our population, the decay of LD varied across chromosomes of both sub-genomes with an average of 4.5 Mb for the A sub-genome and 4.6 Mb for the B sub-genome (Supplementary Figure 1). The average LD of the A sub-genome (*r*^2^ = 0.39) and B sub-genome (*r*^2^ = 0.40) was not divergent (*p* = 0.6961) which may indicate comparable selection pressure for important agronomic traits in the two sub-genomes of the durum panel. Chromosomes 2A and 4B had the slowest in the rate of LD decay (∼8 Mb) (Supplementary Figure 1) indicating that the mapping resolution on these chromosomes is low although chromosome 4B did not contain any significant MTAs. Studies on LD patterns in durum wheat were reported using low density markers (Letta et al., 2013, 2014) and some using relatively high density markers (SNP markers) (Mengistu et al., 2016; Chao et al., 2017) on worldwide durum wheat collections and landraces. Although the decay of LD in these studies was described in genetic distances which may be difficult to compare with our results, it was reported that LD can vary from 5 cM in diverse breeding lines to 20 cM in worldwide collections (Chao et al., 2017).

### Comparison of Significant Markers With Previous Studies

The comparison of our results with previous linkage mapping and association mapping studies on resistance to multiple races in East Africa and few others from different regions of the world validated many of the significant MTAs identified in our study (Supplementary Tables 2–8). Many of the MTAs in our study were consistent across two to five seasons (Table 2 and Supplementary Table 8) indicating the reliability of the results of our GWAS analyses and effectiveness of resistance to multiple stem rust races though seasonal variability in the frequency of race compositions is inevitable in the respective regions of evaluation as indicated in the differences in the mean responses of the population across the five environments (Table 1).

Three significant markers (95, 144, and 485 Mb) were identified on chromosome 1A (Figure 3 and Supplementary Figure 2). Markers *IWB57448* and *IWA8622* reported by Bajgain et al. (2015b), one of the flanking markers of a QTL identified by Bhavani et al. (2011) (*wPt-734078*), and markers *IWA2057* and *IWA5702* reported by Gao et al. (2017) tagging *Sr31* for resistance to TTTTF and TRTTF were not close to the markers we identified on 1A. These three markers were in linkage equilibrium. The MTAs at 95 and 485 Mb may represent novel QTL while the 144 Mb region was on the threshold line (FDR adjusted *p* = 0.04) (Figure 3) which makes this association unreliable and it could be false positive.

On chromosome 1B, four significant MTAs were detected (Figure 4 and Supplementary Figure 2). The marker at 546 Mb is close to *barc61* (2.7 Mb away) reported by Letta et al. (2014) for seedling resistance of durum accessions to TRTTF, TTTTF, and TTKSK while the marker at 620 Mb region is 2.2 Mb away from *barc81* reported by the same author for seedling resistance to races TTTTF and TTKSK and may tag the same QTL. The MTA at 183 Mb is 3 Mb away from *IWB9794* reported by Bajgain et al. (2015b) for seedling resistance of spring wheat to TRTTF, but this marker had an FDR adjusted *p*-value close to threshold (0.045) while the MTA at 587 Mb is 1.5 Mb away from *IWB40197* reported by Edae et al. (2018) for seedling resistance of spring wheat to race QFCSC likely representing the same locus. Chromosome 1BL is known to harbor the adult plant leaf rust resistance gene *Lr46*, that is tightly linked to the APR gene for stem rust, *Sr58.* However, one of the flanking markers to *Lr46, wmc44* and the same marker reported by Letta et al. (2014) for seedling resistance of durum wheat to TTTTF and JRCQC are further away from the marker we detected. Screening of the lines with the KASP marker designed for *Lr46* (*CIMwMAS0085*)^2^ indicated that 46% of the lines are expected to carry *Lr46/Sr58*, however, this locus was not significant in our study. This may be because of the confounding effect of major gene resistances in our population as the lines were evaluated for field response regardless of their seedling response or the *Lr46* marker may not be predictive.

We identified seven significant MTAs on chromosome 2A (Figures 3, 4). The MTA at 20 Mb detected in ETOS18 is close to *wPt-5839* (386 kb away) reported by Letta et al. (2014) for seedling resistance of durum wheat accessions to TRTTF, TTTTF, and TTKSK likely representing the same QTL. No known marker close to the QTL at 67, 78, 135, 728, and 770 Mb regions was reported previously. Therefore, these five markers are representing putatively novel loci. One MTA at 699 Mb with an FDR adjusted *p-*value close to the threshold (0.049) is likely to be false positive (Supplementary Table 3). Chromosome 2A is known to host *Sr21* and *Sr38* transferred to hexaploid wheat from *Triticum monococcum* and *Triticum ventricosum*, respectively (Singh et al., 2011; Chen et al., 2018). About eight lines in the panel possess *Sr38* (Ammar, personal communication, 2020) but it is unlikely to be detected due to the MAF below the threshold. Both *Sr21* and *Sr38* are ineffective against the *Ug99* lineages (predominant in Kenya), TKTTF and JRCQC (predominant in Ethiopia) (Olivera et al., 2015).

On chromosome 2B, four significant markers were identified (Figures 3, 4). The MTA at 759 Mb is close (8 Mb away) to marker *wmc361* reported by Letta et al. (2013) and Yadav et al. (2015) likely representing the region of *SrWeb/Sr9h. SrWeb/Sr9h* is effective against Ug99 (Jin et al., 2007; Rouse et al., 2014a) and this MTA (759 Mb) was identified in KNMS19 where Ug99 is predominant. The MTA at 780 Mb is 7.4 Mb away from *wmc356* reported by the same author for APR of durum wheat to Ug99 that co-locates with the region of *Sr28/Sr16*. Several markers were reported by a number of authors on chromosome 2B (Letta et al., 2013, 2014; Yu et al., 2014; Bajgain et al., 2015b; Chao et al., 2017; Gao et al., 2017; Edae et al., 2018), but none are close to the remaining two significant markers. The 456 Mb region may represent a novel locus but identified in one season only while the 569 Mb region had an FDR adjusted *p-*value close to the threshold (0.046) which may indicate unreliable association (Supplementary Tables 2, 3). Chromosome 2B is known to carry the alleles of *Sr9* (*Sr9a*, *Sr9b*, *Sr9d*, *Sr9e*, *Sr9f*, *Sr9g*, *SrWeb/Sr9h*), *Sr28, Sr36*, and *Sr16.* Among the seven alleles of *Sr9*, five of them are ineffective against Ug99 while *Sr9e* is reported to be inconclusive (Jin et al., 2007; Rouse et al., 2014a). *Sr9a*, *Sr9d*, *Sr9e*, and *Sr9g* are ineffective against JRCQC and TKTTF (Olivera et al., 2012). *Sr28* is effective against Ug99 but *Sr16* is not (Rouse et al., 2014a). *Sr36* confers resistance to TTKSK and TTKST (Jin et al., 2007; Rouse et al., 2014a) but ineffective to TTTSK (Ug99 lineage), TTRTF and TKTTF (Jin et al., 2009; Olivera et al., 2012, 2015) and this gene was transferred to common wheat from *Triticum timopheevi* (Jin et al., 2009) and it is unlikely to exist in the durum wheat panel.

Three significant markers (9, 313, and 344 Mb) were identified on chromosome 3A (Figures 3, 4). Markers *wPt6854* and *barc12* reported by Letta et al. (2013) are close to the marker at 9 Mb (5 Mb away) indicating that this marker may represent the same region though identified in one season only. Markers *wmc264, wPt-8203*, *barc1177*, and *wmc388* reported by Letta et al. (2013, 2014) are further away from the remaining two markers on 3A. So, the MTAs at 313 and 344 Mb may represent novel loci for field resistance to *Pgt* races in Ethiopia albeit both were identified in one season. Chromosome 3A is known to host *Sr27* and *Sr35*, and both are effective against Ug99 (Jin et al., 2007; Rouse et al., 2014a). *Sr35* was transferred from *Triticum monococcum* to common wheat (Zhang et al., 2010) while *Sr27* was transferred from rye to common wheat (Jin et al., 2009; Letta et al., 2013). None of these wild relative-derived genes are known to have been introgressed into the CIMMYT durum germplasm.

Five significant MTAs were identified on chromosome 3B (Supplementary Tables 2, 3). Markers *wPt-0365* and *wPt-6802* reported by Yu et al. (2014) tagging *Sr12* is 14 Mb away from the MTA at 724 Mb. Flanking markers of *Sr12* (*wPt-0544* and *wPt-6047*) reported by Rouse et al. (2014b) are further away from the 724 Mb locus. However, this marker lies between the regions reported by Yu et al. (2014) and Rouse et al. (2014b) indicating that it could be representing *Sr12*. Rouse et al. (2014b) reported that *Sr12* confers resistance to Ug99 (TTKSK) at adult plant stage when combined with other resistance loci in a QTL study of Thatcher/McNeal RIL population. Although no significant interaction was observed with any of the known *Sr* genes postulated in our GWAS result, significant interactions were observed between the marker at 724 Mb region and QTL on chromosome1B (at 620 Mb) (*p* = 0.020903) and 5B (688 Mb) (*p* = 0.013911) for resistance to multiple races in Ethiopia and Kenya, respectively. The MTA at 9 Mb region that was consistently identified in four of the five testing environments using MLM was not close to any of the previously reported markers suggesting that it may represent a novel locus unidentified by FarmCPU (Supplementary Table 8). The remaining three MTAs were identified in one season only. One of the three markers at 213 Mb region had FDR adjusted *p-*value close to the threshold (0.042) (Supplementary Table 3) and this marker is close to *wmc43* (4.5 Mb away) reported by Letta et al. (2014) but less reliable. The MTA at 55 Mb region is 14 Mb away from *wPt-6945* reported by Yu et al. (2011) likely identified the same region. No known marker close to the MTA at 38 Mb region was reported previously and this marker had an FDR adjusted *p-*value close to the threshold (0.036) which makes this association less reliable. The short arm of chromosome 3B is known to harbor the known APR gene, *Sr2* but this gene is not present in the CIMMYT durum germplasm as confirmed by the screening of the panel using KASP marker designed for *Sr2* (*Sr2_ger93p*, Mago et al., 2011) and the absence of the pseudo black chaff trait (morphological marker for *Sr2*) in any of the lines in greenhouse and field.

Two significant MTAs (619 and 651 Mb) were identified on chromosome 4A (Supplementary Tables 3–7). The region at 651 Mb is 1.5 Mb away from one of the flanking marker (*wPt -5857*) of a QTL on chromosome 4AL reported by Yu et al. (2014) on Ug99 resistance consensus map of wheat and likely identified the same locus. None of the markers reported by Letta et al. (2014), Yu et al. (2011, 2014), and Bajgain et al. (2015b) are close to the marker at 619 Mb region indicating that this marker is likely representing a novel resistant locus. Chromosome 4A hosts the alleles of *Sr7* (*Sr7a*, *Sr7b*). *Sr7a* confers resistance against race TKTTF (Olivera et al., 2015) whereas *Sr7b* is effective against race JRCQC (Olivera et al., 2012).

Two significant markers were identified on chromosome 5A at 8 and 35 Mb regions (Figures 3, 4). Markers *IWA1062*, *IWA5040*, and *IWA5368* reported by Chao et al. (2017) for seedling resistance of durum wheat accessions to races TTRTF, JRCQC, and bulk races in Debre Zeit, Ethiopia; *IWB47184*, *IWA2224, IWA2836*, and *IWB34927* reported by Bajgain et al. (2015b) for APR of spring wheat to Ug99 and seedling resistance to TKTTF; *barc165* reported by Letta et al. (2014) for seedling resistance of durum wheat accessions to race JRCQC are not close to the markers we detected on 5A. These two markers likely represent novel loci for field resistance to multiple races in Ethiopia and Ug99 lineages in Kenya, but they were identified in one season.

On chromosome 5B, seven significant MTAs were identified (Figures 3, 4). Bansal et al. (2014) reported markers *sun209* and *sun479* flanking *Sr49* which is effective against all the races in Australia. The MTA at 691 Mb co-locates with *sun479* (530 kb away) while 692 Mb region co-locates with *sun209* (485 kb away). These two markers (691 Mb and 692 Mb) were consistent across four of the five seasons though limited by the low MAF (0.053 on average) which indicates that this gene is rare in the panel (Supplementary Table 2). The 691 Mb locus was detected for resistance to TKTTF at the seedling stage (manuscript on preparation) indicating that these two markers are representing an all stage multiple-race specific resistance gene likely *Sr49*. Bhavani et al. (2011) reported flanking markers *wPt0750* and *wPt5896* on chromosome 5BL in biparental mapping (PBW343/Juchi) for APR to Ug99 in hexaploid wheat. The MTA at 581 Mb identified in KNMS19 using both models, is close to these flanking markers (∼5–6 Mb away) and was detected at the adult plant stage in Kenya only. Hence, this marker is likely tagging the same locus as Bhavani et al. (2011). One of the flanking markers (*wPt8604)* of a QTL reported by Yu et al. (2014) on the Ug99 resistance consensus map of wheat is 7 Mb away from two MTAs identified at 13 and 12 Mb regions likely representing the same QTL (Figures 3, 4). A number of markers have been reported by several authors on chromosome 5B (Letta et al., 2013; Bansal et al., 2014; Yu et al., 2014; Bajgain et al., 2015a; Mago et al., 2015; Chao et al., 2017) but none of them are close to the markers at 688 Mb and 671Mb regions identified in ETOS18 and KNMS19, respectively (Supplementary Tables 2, 3). The long arm of chromosome 5B hosts the adult plant resistance gene *Sr56* and an all stage resistance gene *Sr49* (Bansal et al., 2014, 2015). Both durum and common wheat can have *Sr56*. However, markers linked to *Sr56* reported by Bansal et al. (2014, 2015) are further away from the MTAs at 671Mb and 688 Mb. Therefore, these two markers may represent novel loci for field resistance to races in Kenya and Ethiopia although detected in only one season.

On chromosome 6A, 52 significant MTAs representing five QTL mapped the regions of previously reported loci and novel loci (Supplementary Tables 2–7). None of the markers reported by Letta et al. (2013, 2014), Bajgain et al. (2015b), and Chao et al. (2017) are close to the MTA at 592 kb region. Markers *IWA7913*, *IWA7006*, *IWB23519* reported by Bajgain et al. (2015b) and Gao et al. (2017) for seedling resistance of spring wheat to race TRTTF and BCCBC are very close to an MTA at 4 Mb region (∼3–5 kb away). Guerrero-Chavez et al. (2015) reported that these markers are linked to *Sr8a.* Marker *IWB72958* reported by Nirmala et al. (2017) is linked to *Sr8155B1* in durum wheat that is effective against TTKST and TRTTF and this marker is ∼4.8 kb away from the marker at 4 Mb region. Moreover, *Sr8155B1* was reported effective against races in Njoro, Kenya but not effective against races in Debre Zeit, Ethiopia (Nirmala et al., 2017). Similarly, the MTA at 4 Mb region was identified for adult plant resistance of durum lines in Kenya only where race TTKST is predominant. This indicates that the MTA at 4 Mb likely maps the region of *Sr8155B1*. The marker at 592 kb was in strong LD (*r*^2^ = 0.89) with the 4 Mb region. However, the 592 kb region was associated with resistances to races in Ethiopia where the virulent races to *Sr8155B1* (JRCQC and TTKSK) are predominant indicating that this MTA may represent a new allele at the *Sr8* locus or a novel gene linked to the *Sr8* locus. The high LD between these two loci may indicate limited recombination rate in the regions or the resistance alleles might be selected together. Markers *wPt1742* and *wPt1377* reported by Letta et al. (2013) for field resistance of durum wheat accessions to Ug99 are close to (∼765 and 845 kb away). An MTA at 1.4 Mb identified for field resistance in ETOS18 (Supplementary Table 2). Markers *IWA272*, *IWB64917*, *IWB64918*, *IWB5029*, *IWB35595*, *IWB43808*, *IWB72956* reported by Bajgain et al. (2015b) for seedling resistance of spring wheat to TRTTF are 1 Mb away from the MTA at 1.4 Mb indicating that this MTA likely maps the region of *Sr8a* though identified in one season only. It is known that the short arm of chromosome 6A hosts the alleles of *Sr8 (Sr8a and Sr8b*) and *Sr8a* confers resistance to the predominant races in Ethiopia, TRTTF (Jin et al., 2007; Nirmala et al., 2017) and JRCQC (Olivera et al., 2012) but both alleles are ineffective against TTKSK and TTKST at seedling and adult plant stage (Jin et al., 2007). No known marker close to the markers at 28, 189, and 334 Mb regions of chromosome 6A (Supplementary Tables 3–7) was previously reported. The MTAs at 28 Mb and 334 Mb regions likely represent new loci whereas the one at 189 Mb was identified in one season only and is on the FDR threshold line (Supplementary Figure 2) which makes this association less reliable. All the significant markers identified on chromosome 6A from 606 to 615 Mb regions collocate with markers tagging *Sr13* region including *CD926040* and *barc104* reported by several authors (Simons et al., 2011; Letta et al., 2013, 2014), *IWA4918* reported by Chao et al. (2017), *IWA7495* reported by Gao et al. (2017) for seedling and adult plant resistance to multiple *Pgt* races, and the flanking markers of *Sr13, CJ671993*, and *CJ641478* reported by Zhang et al. (2017). Therefore, the MTAs extended from 606 Mb to 615 Mb regions of chromosome 6A likely represent *Sr13*/alleles. It is known that *Sr13* is an all stage resistance gene to the Ug99 lineages. The higher percentage of lines (69%) carrying *Sr13* on marker screening may indicate the wide usage of this gene in CIMMYT durum wheat breeding program. This result is proven by the higher frequency (27–85%) of the favorable alleles at the *Sr13* locus. However, more than one allele is expected as indicated in the differences in allele frequencies and the LD between nearby markers (Supplementary Tables 3–7 and Figure 5). The alleles, *Sr13a* and *Sr13c* confer resistance to the most virulent races of durum wheat including JRCQC and TTRTF and to the Ug99 lineages (Olivera et al., 2019, Olivera, personal communication, 2020) while *Sr13b* confers resistance against TTKSK, TKTTF, TRTTF (Zhang et al., 2017; Randhawa et al., 2018) but is ineffective against JRCQC and TTRTF (Zhang et al., 2017). Three MTAs, at 611 and 612 Mb (two at 612 Mb) identified as the most significantly associated markers for field resistance to multiple races (Supplementary Tables 2, 3) in the different testing environments were also identified at the seedling stage (manuscript on preparation). These markers could potentially be used to identify the different alleles of *Sr13* although further study and validation on different populations will be needed. In some cases, the LD between the significant markers identified on chromosome 6AL at the *Sr13* region was slightly below the threshold or weak (Figure 5), suggesting that the region could be a recombination hotspot which can lead to low intra-chromosomal LD.

On chromosome 6B, six significant MTAs representing four putative QTL were identified (Supplementary Tables 2, 5). Several markers (*IWB24880, IWB46893, IWB48548, IWB71190, IWB47075*) reported by Bajgain et al. (2015b) for seedling resistance of spring wheat to TKTTF, and *IWB35697* for adult plant resistance to Ug99 in Ethiopia and Kenya, are close to the MTA at 692 Mb (229 kb–2 Mb away). Marker *KASP_6BL_IWB72471* reported by Nirmala et al. (2016) as a predictive marker for *Sr11* is 2 Mb away from this marker indicating that it is likely mapping the *Sr11* locus. However, *Sr11* is ineffective against TTKSK, JRCQC, and TRTTF at the seedling stage and is effective against TKTTF (Jin et al., 2007; Olivera et al., 2012) which is among the predominant races in Ethiopia where the association was identified (ETOS19). It is known that residual effects of ineffective major gene resistances are among the possible mechanisms of field quantitative resistance. Two MTAs at 686 and 687 Mb region were in strong LD (*r*^2^ = 0.64) and represent the same QTL (Supplementary Table 5). Several markers reported by Bajgain et al. (2015b) are close to these two markers. The closest markers, *IWA4245* and *IWA4246* are 502 kb away from the 686 Mb locus while *IWB59175.2* is 196 kb away from 687 Mb region indicating that the two markers may represent the same region as the one reported by Bajgain et al. (2015b). None of the markers reported by Bajgain et al. (2015b), and markers *wPt1541, barc79, wPt4930, wPt5333*, and *wPt5037* reported by Yu et al. (2014) are close to the MTAs at 31 Mb, 30 Mb and 666 Mb regions. The two markers at 31 Mb and 30 Mb regions were in LD (*r*^2^ = 0.33) indicating that they represent the same QTL in the short arm of 6B which is likely novel and the MTA at 666 Mb region could also be representing a novel locus (Supplementary Table 2).

We identified 60 significant MTAs on chromosome 7A (Supplementary Tables 2–7). The markers that extended from 668 to 727 Mb were in LD and may represent a single QTL (Figure 6). The 700 and 717 Mb regions were identified in multiple seasons (Supplementary Table 8) suggesting that these markers are tagging a multiple-race resistance locus. Markers *IWB5070, IWB1874, IWB4830*, and *IWB62560* reported by Bajgain et al. (2015b) for adult plant resistance of spring wheat to Ug99 are 2 Mb away from the MTA at 700 Mb region. Marker *IWB48466* reported by the same author is 5 Mb away from the MTA at 717 Mb region. Marker *IWA2270* reported by Chao et al. (2017) for resistance of durum wheat accessions to race TTTTF tagging the *Sr22* locus co-locates with the MTA at 673 Mb (∼5 kb away). These three markers (673, 700, and 717 Mb) were in moderate to strong LD (*r*^2^ = 0.37–0.83) indicating that these MTAs are representing the region of *Sr22.* This gene confer resistance to TTKSK (Jin et al., 2007), JRCQC and TRTTF (Olivera et al., 2012) and transferred from *T. monococcum* (Olson et al., 2010). The resistance allele at the *Sr22* locus is probably rare in the study population as observed in the frequency of the favorable alleles (Supplementary Tables 2–7). Some of the lines in the panel (∼10 lines) poses *Sr25* (Ammar, personal communication, 2020). However, it is unlikely to identify the *Sr25* locus due to MAF below the threshold (0.05). *Sr25* and *Sr22* come with severe yield penalties in durum wheat (Ammar, personal communication, 2020). So, breeders should be prepared to conduct several cycles of selection to use these gene with minimal to no performance penalties. None of the markers listed earlier including markers *IWA7200* reported by Chao et al. (2017), *barc70* and *wmc479* reported by Letta et al. (2013), *Xbarc121* reported by Yu et al. (2014) are close to the MTAs at 43, 117, 139, and 285 Mb regions of chromosome 7A and these MTAs were identified in one season only. Moreover, two of the regions had FDR adjusted *p-*value close to the threshold (Supplementary Table 2, 5) indicating that these loci could be false positives.

On chromosome 7B, we identified five significant MTAs (Supplementary Tables 2–7). The MTA at 717 Mb is 8 Mb away from *IWB47548* and *IWA4175* reported by Bajgain et al. (2015b) for adult plant resistance of spring wheat to Ug99 indicating that this MTA is likely representing the same locus. The MTA at 644 Mb is 7 Mb away from an SSR marker linked to *Sr17* (*wmc517*) reported by Letta et al. (2014) for seedling resistance of durum wheat accessions to races TTTTF and TTKSK. So, this MTA (644 Mb) and an MTA at 622 Mb (LD, *r*^2^ = 0.64) likely represent *Sr17*. The consistency of these two MTAs across three seasons may indicate the reliability of association although the resistance allele at this locus is rare in the population (only 7% of the lines/19 lines carry the resistance allele on average). Markers *wmc182, wmc517, wPt1715, wPt4298, wPt7191, wPt4045* reported by Letta et al. (2013), and marker *wPt1149* reported by Yu et al. (2014) are further away from the MTA at 46 Mb region and this region is likely novel. The MTA at the 707 Mb is 2 Mb away from *IWB47548* and *IWB4175* reported by Bajgain et al. (2015b), but the FDR adjusted *p-*value was close to the threshold (0.047) which makes this association less reliable. We identified nine significant MTAs on an unknown chromosomal location (Supplementary Tables 2–7). Four of the nine MTAs were identified in one season only while the remaining five were identified in three to five seasons and we were unable to find a location for these markers.

Overall, a number of lines were consistently resistant across the five seasons in the two hotspot regions (Ethiopia and Kenya) and can be used as sources of resistance to multiple stem rust races in East Africa. Once these lines are evaluated for agronomic performance, combining more resistance alleles and/or genes to the best performing lines may increase durability of resistance to potentially emerging races. Among a total of 160 significant MTAs identified using MLM and FarmCPU with known chromosomal locations and grouped to 42 QTL, 21 QTL are putatively novel and the remaining 21 are mapped to previously reported regions. The regions representing *Sr12, Sr13/*alleles, *Sr17*, *Sr22*, and *Sr49* are among the known resistant genes consistent in two to five seasons for resistance to multiple-races in East Africa. *Sr13* was more frequent in the population while *Sr12*, *Sr17*, *Sr22*, and *Sr49* were less frequent. Novel loci consistent across multiple seasons were also identified on chromosomes 3B, 4A, 6A, and 6B and the resistance alleles at the loci on chromosomes 3B, 4A, and 6A were less frequent. Therefore, breeders should try to retain these rare genes/alleles during the selection process in future breeding plans. The markers identified in the current study once validated and optimized for high-throughput platforms, can be used in marker-assisted selection to combine sources of resistance to stem rust in durum wheat. The information on the available sources of resistance in this panel is also useful for future deployment of the resistance sources in durum wheat breeding programs. The region of *Sr13* on chromosome 6AL is wider and the extent of LD is complex. Therefore, allelism tests and further studies on the validation of potential allele specific markers for *Sr13* are needed.

## Data Availability Statement

The datasets presented in this study can be found in online repository. The name of the repository/repositories and accession number(s) can be found below: https://figshare.com/s/5b5378408568ead07846/10.6084/m9.figshare.12949295; https://figshare.com/s/40b2f7c7be1ce931a4d2/10.6084/m9.figshare.13203194.

## Author Contributions

SM, MS, and MA designed the research. KA developed and assembled the mapping population, participated in experimental design, and provided seed to each evaluation site. SM, AD, and MR conducted the field experiments. GB-G and BW carried out genotyping and SNP calling. SM conducted the data analysis and drafted the manuscript. All co-authors read and reviewed on the manuscript.

## Conflict of Interest

The authors declare that the research was conducted in the absence of any commercial or financial relationships that could be construed as a potential conflict of interest.

## Footnotes

1 https://www.integratedbreeding.net

2 https://www.integratedbreeding.net

## Supplementary Material

Supplementary Figure 1 Scatter plot of squared allele-frequency correlations (r^2^) vs. physical distance (Mb) between pairs of markers indicating the decay of linkage disequilibrium (LD) across the 14 chromosomes of the durum wheat panel.

Supplementary Figure 2 Manhattan and QQ-plots of GWAS results of field resistance of durum wheat lines to multiple races in Ethiopia across three seasons identified using MLM.

Supplementary Figure 3 Manhattan and QQ-plots of GWAS results of field resistance of durum wheat lines to multiple races in Kenya across two seasons identified using MLM.

Supplementary Table 1 Mean coefficient of infection of lines positive to *Sr13* and *Lr46/Sr58* marker screening with multiple-race resistance at the adult plant stage.

Supplementary Table 2 Lists of SNPs significantly associated with field resistance to East African *Pgt* races across five seasons identified using FarmCPU.

Supplementary Table 3 Lists of SNPs significantly associated with field resistance to *Pgt* races in Ethiopia during the off-season 2018 (ETOS18) identified using MLM.

Supplementary Table 4 Lists of SNPs significantly associated with field resistance to *Pgt* races in Ethiopia during the main-season 2018 (ETMS18) identified using MLM.

Supplementary Table 5 Lists of SNPs significantly associated with field resistance to *Pgt* races in Ethiopia during the off-season 2019 (ETOS19) identified using MLM.

Supplementary Table 6 Lists of SNPs significantly associated with field resistance to *Pgt* races in Kenya during the main-season 2018 (KNMS18) identified using MLM.

Supplementary Table 7 Lists of SNPs significantly associated with field resistance to *Pgt* races in Kenya during the main-season 2019 (KNMS19) identified using MLM.

Supplementary Table 8 Lists of consistent significant markers between testing environments identified using MLM.

Supplementary Table 9 Lists of pedigrees of a panel of durum wheat lines used for the study.

Supplementary Table 10 Information on KASP assays designed for screening lines for the presence of *Sr2*, *Sr13*, and *Lr46/Sr58*.

## Abbreviations

APR: Adult plant resistance
BLUPs: Best linear unbiased predictions
CI: coefficient of infection
CIMMYT: International Maize and Wheat Improvement Center
CMLM: Compressed Mixed Linear Model
DArT: Diversity arrays technology
FarmCPU: Fixed and random model Circulating Probability Unification
FDR: False Discovery Rate
GBS: Genotyping-by-sequencing
GAPIT: Genomic Association and Prediction Integrated Tool
GID: Genotype identification number
GWAS: Genome-wide association analysis
IT: Infection type
KASP: kompetitive allele-specific PCR
LD: Linkage Disequilibrium
LMM: Linear mixed model
LOESS: Locally estimated scatterplot smoothing
MAF: Minor Allele Frequency
MLMM: Multi-locus Mixed Linear Model
MTAs: Marker trait associations
PCA: principal component analysis
SNP: Single Nucleotide Polymorphism
QTL: quantitative trait locus/loci
Q-Q: quantile-quantile
SSRs: simple sequence repeats.
