# Supplementary figures and images for: Multiple-Race Stem Rust Resistance Loci Identified in Durum Wheat Using Genome-Wide Association Mapping
Source: Front Plant Sci. 2020 Dec 17;11:598509. doi: 10.3389/fpls.2020.598509 (PMC7773921; doi:10.3389/fpls.2020.598509)

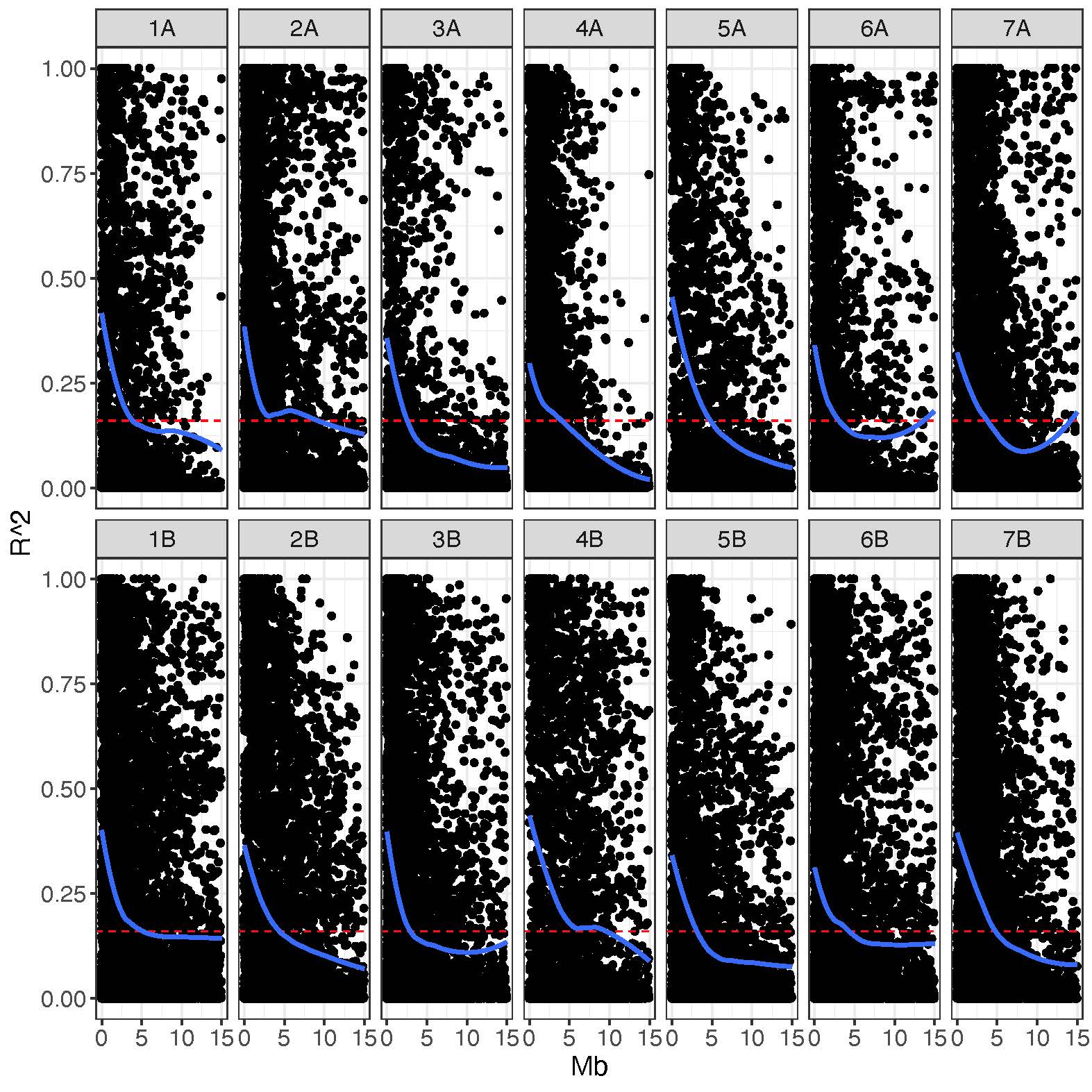

Supplement: Supplementary Figure 1 — Scatter plot of squared allele-frequency correlations (r2) vs. physical distance (Mb) between pairs of markers indicating the decay of linkage disequilibrium (LD) across the 14 chromosomes of the durum wheat panel. [file Image_1.jpg]

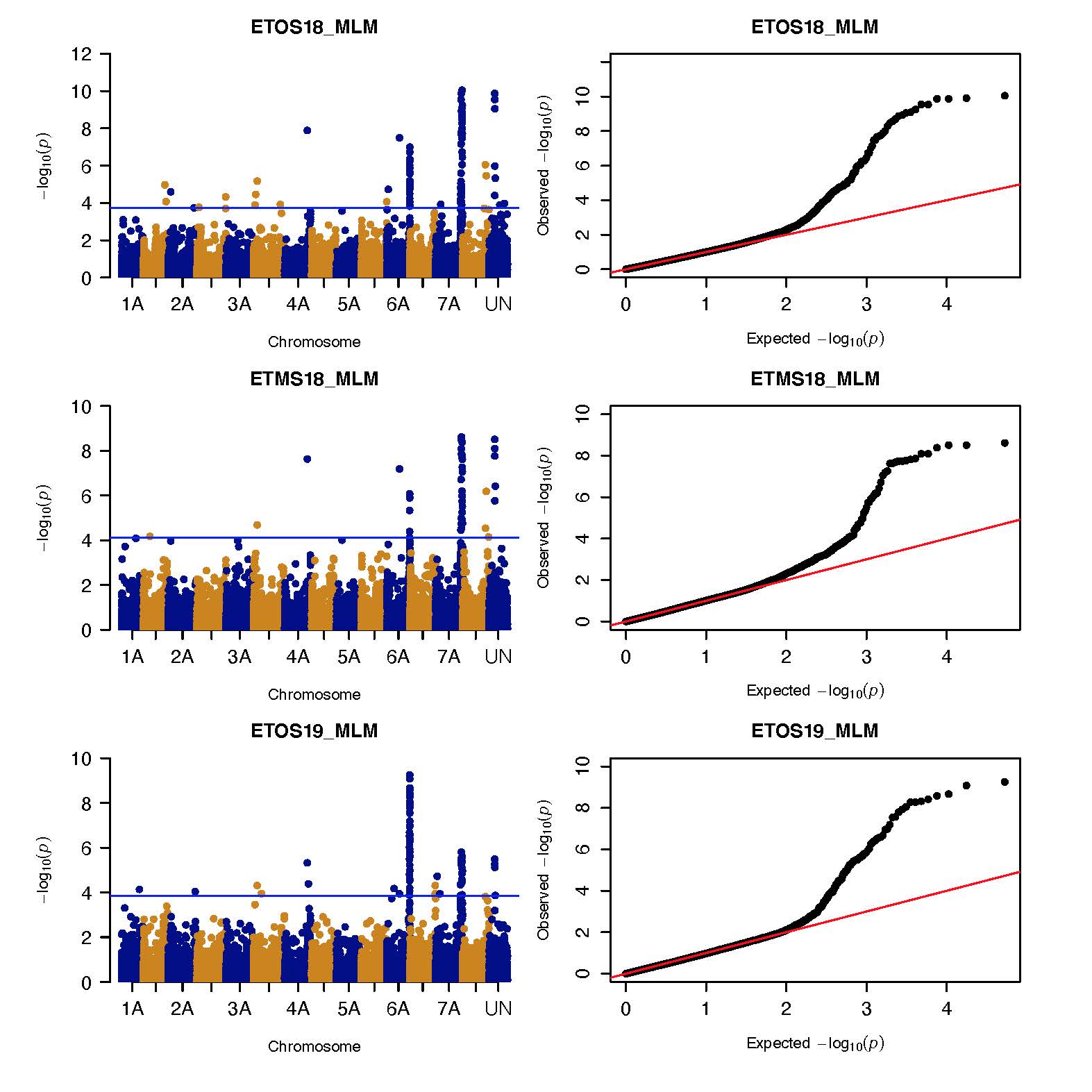

Supplement: Supplementary Figure 2 — Manhattan and QQ-plots of GWAS results of field resistance of durum wheat lines to multiple races in Ethiopia across three seasons identified using MLM. [file Image_2.jpg]

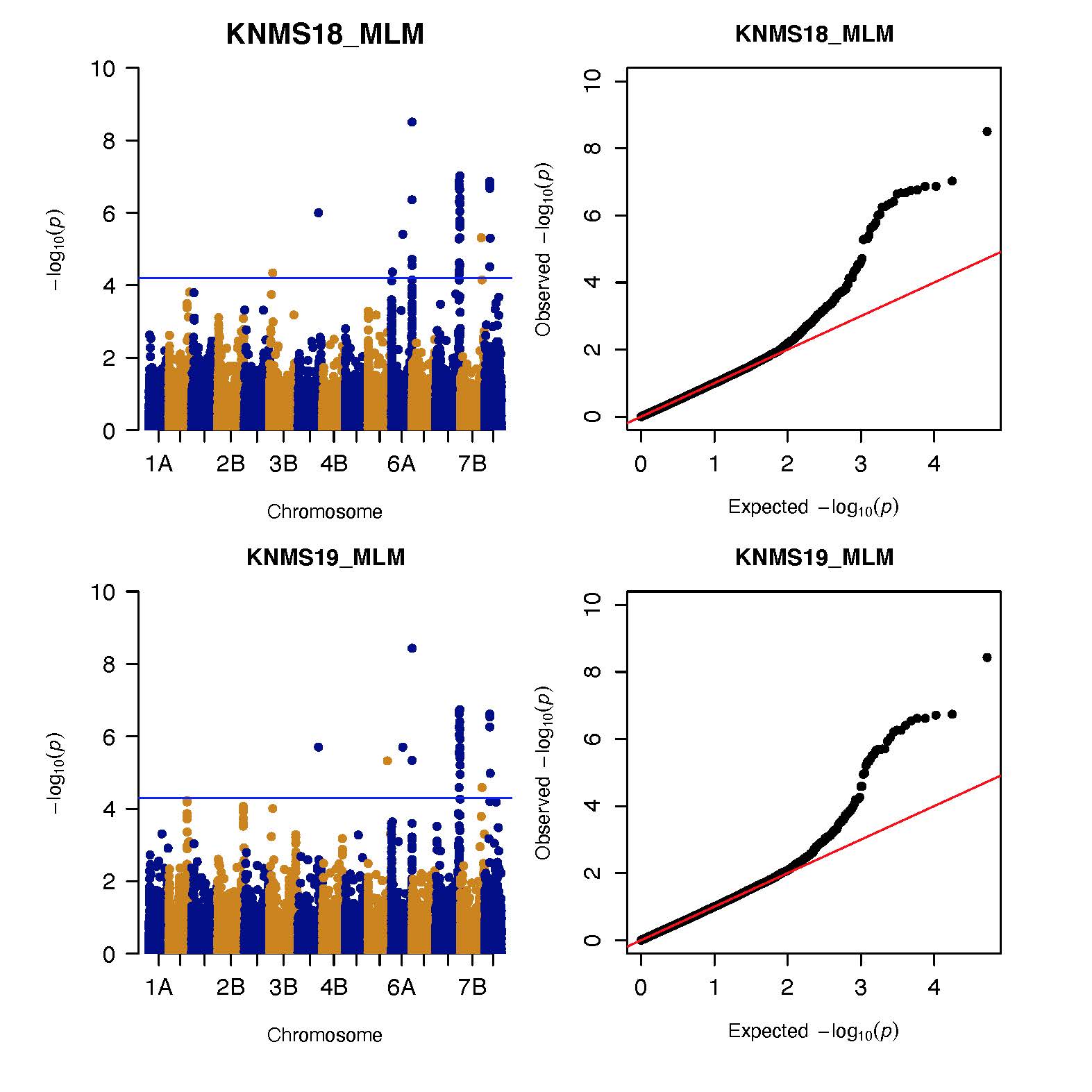

Supplement: Supplementary Figure 3 — Manhattan and QQ-plots of GWAS results of field resistance of durum wheat lines to multiple races in Kenya across two seasons identified using MLM. [file Image_3.jpg]
