# Supplementary material for: Multiple-Race Stem Rust Resistance Loci Identified in Durum Wheat Using Genome-Wide Association Mapping
Source: Front Plant Sci. 2020 Dec 17;11:598509. doi: 10.3389/fpls.2020.598509 (PMC7773921; doi:10.3389/fpls.2020.598509)
Supplement: Supplementary Table 1 — Mean coefficient of infection of lines positive to Sr13 and Lr46/Sr58 marker screening with multiple-race resistance at the adult plant stage. [file Table_1.docx]

| Supplementary TABLE 1. Mean coefficient of infection of lines positive to *Sr13* and *Lr46/Sr58* marker screening with multiple-race resistance at the adult plant stage. | | | | | |
| --- | --- | --- | --- | --- | --- |
| Origin GID | ETOS18 | ETMS18 | ETOS19 | KNMS18 | KNMS19 |
| 7145228 | 25.5 | 12.0 | 22.5 | 10.0 | 15.0 |
| 7145451 | 43.0 | 47.5 | 27.8 | 5.0 | 21.0 |
| 7145526 | 40.0 | 38.3 | 23.5 | 15.0 | 13.5 |
| 7145583 | 31.3 | 13.5 | 9.8 | 7.5 | 3.8 |
| 7145599 | 39.0 | 23.0 | 12.5 | 13.5 | 18.0 |
| 7145651 | 21.8 | 5.8 | 3.3 | 4.8 | 6.0 |
| 7145664 | 27.0 | 15.0 | 27.0 | 7.5 | 16.5 |
| 7145707 | 8.8 | 7.0 | 6.0 | 18.0 | 5.5 |
| 7145713 | 22.5 | 27.0 | 11.3 | 40.5 | 22.5 |
| 7145733 | 48.5 | 7.5 | 14.0 | 13.5 | 12.0 |
| 7145764 | 19.5 | 12.0 | 21.0 | 5.5 | 12.0 |
| 7145770 | 36.0 | 30.0 | 13.0 | 18.0 | 10.5 |
| 7145771 | 11.5 | 11.0 | 7.5 | 6.5 | 5.5 |
| 7145779 | 31.5 | 13.5 | 15.0 | 7.0 | 10.0 |
| 7145795 | 32.0 | 18.0 | 16.5 | 2.5 | 21.0 |
| 7145800 | 38.0 | 18.0 | 13.8 | 13.0 | 21.0 |
| 7383281 | 22.0 | 10.0 | 18.0 | 12.0 | 12.0 |
| 7383291 | 45.0 | 40.5 | 34.0 | 17.0 | 12.0 |
| 7383456 | 38.0 | 25.5 | 36.0 | 3.8 | 7.5 |
| 7383862 | 40.5 | 24.0 | 21.5 | 12.8 | 24.0 |
| 7384046 | 14.0 | 11.5 | 12.3 | 5.3 | 12.0 |
| 7384063 | 27.0 | 16.0 | 9.8 | 13.0 | 21.0 |
| 7384071 | 20.3 | 9.0 | 17.5 | 6.8 | 13.5 |
| 7384072 | 36.0 | 10.5 | 7.5 | 10.5 | 39.0 |
| 7384079 | 31.5 | 11.8 | 10.8 | 26.0 | 18.0 |
| 7384096 | 38.0 | 15.0 | 20.0 | 12.0 | 15.0 |
| 7406259 | 36.0 | 0.0 | 5.0 | 5.0 | 9.0 |
| 7406303 | 21.8 | 10.3 | 10.0 | 8.0 | 15.0 |
| 7406313 | 31.5 | 33.5 | 27.0 | 5.5 | 12.0 |
| 7406340 | 43.0 | 11.0 | 20.3 | 5.5 | 7.5 |
| 7406449 | 38.0 | 16.0 | 20.0 | 5.5 | 2.3 |
| 7406486 | 30.0 | 14.0 | 13.5 | 3.3 | 2.8 |
| 7406533 | 45.0 | 11.0 | 20.5 | 8.5 | 24.0 |
| 7406594 | 27.0 | 12.5 | 10.0 | 2.3 | 10.3 |
| 7406684 | 40.5 | 21.0 | 18.3 | 13.0 | 34.5 |
| 7406808 | 34.0 | 9.8 | 24.0 | 8.5 | 11.0 |
| 7406899 | 24.3 | 18.5 | 12.0 | 7.5 | 10.5 |
| 7407025 | 31.5 | 8.5 | 6.3 | 9.0 | 7.5 |
| 7407092 | 36.0 | 23.5 | 7.5 | 18.0 | 15.0 |
| 7407117 | 24.8 | 19.0 | 13.3 | 1.0 | 3.0 |
| 7407174 | 27.0 | 19.0 | 27.0 | 1.0 | 9.0 |
| 7407242 | 31.5 | 26.0 | 5.8 | 5.5 | 16.5 |
| 7407561 | 31.5 | 9.8 | 10.0 | 3.0 | 7.5 |
| 7407611 | 36.0 | 34.0 | 18.5 | 7.0 | 21.5 |
| 7407689 | 65.0 | 55.0 | 50.0 | 12.0 | 53.0 |
| 7407740 | 47.5 | 22.0 | 13.8 | 9.0 | 21.5 |
| 7408065 | 38.0 | 25.5 | 16.5 | 13.5 | 25.5 |
| 7408683 | 43.0 | 24.0 | 18.0 | 31.5 | 39.0 |
| 7408843 | 36.0 | 24.0 | 8.3 | 15.0 | 18.0 |
| 7408925 | 36.0 | 32.0 | 17.8 | 34.5 | 45.0 |
| 7409002 | 43.0 | 34.5 | 16.3 | 17.0 | 27.0 |
| 7409071 | 43.5 | 30.0 | 19.5 | 8.0 | 3.8 |
| 7409080 | 43.0 | 26.0 | 10.5 | 7.5 | 16.0 |
| 7409188 | 43.0 | 19.0 | 15.0 | 9.0 | 19.5 |
| 7409275 | 43.0 | 32.5 | 19.5 | 9.0 | 24.0 |
| 7409314 | 55.0 | 43.0 | 16.5 | 37.5 | 36.0 |
| 7409395 | 48.0 | 18.0 | 25.0 | 24.0 | 22.0 |
| 7409461 | 60.0 | 48.0 | 31.5 | 33.0 | 15.0 |
| 7410092 | 45.0 | 30.0 | 29.3 | 12.0 | 11.0 |
| 7410242 | 38.0 | 18.0 | 19.5 | 9.0 | 14.0 |
| 7410277 | 29.5 | 27.0 | 22.5 | 28.5 | 16.0 |
| 7410549 | 55.0 | 20.0 | 26.0 | 12.0 | 12.0 |
| 7410632 | 36.0 | 44.0 | 12.5 | 9.1 | 21.0 |
| 7410795 | 45.0 | 24.0 | 13.8 | 5.0 | 9.0 |
| 7606811 | 55.0 | 25.5 | 20.0 | 7.0 | 13.0 |
| 7606825 | 48.0 | 30.0 | 16.5 | 10.5 | 19.5 |
| 7147237 | 36.0 | 17.5 | 10.5 | 7.0 | 33.0 |
| 7384203 | 43.0 | 36.0 | 18.0 | 25.5 | 15.0 |
| 7405994 | 33.5 | 13.0 | 27.0 | 6.0 | 9.0 |
| 7406012 | 40.0 | 16.5 | 15.8 | 12.0 | 24.0 |
| 7406016 | 43.0 | 9.5 | 25.8 | 10.5 | 18.0 |
| 7406050 | 27.0 | 25.0 | 16.5 | 4.8 | 6.0 |
| 7406069 | 31.3 | 25.5 | 16.5 | 15.0 | 34.5 |
| 6420695 | 6.3 | 4.0 | 8.8 | 4.0 | 1.0 |
| 6420696 | 5.8 | 2.0 | 6.3 | 0.8 | 1.0 |
| 6420697 | 2.0 | 3.0 | 7.0 | 4.0 | 1.3 |
| 6420699 | 19.0 | 3.0 | 11.3 | 2.3 | 1.3 |
| 6420704 | 3.0 | 3.3 | 5.0 | 0.2 | 1.3 |
| 6951168 | 7.0 | 2.0 | 3.3 | 2.3 | 2.8 |
| 5928162 | 5.8 | 10.0 | 6.3 | 6.8 | 6.5 |
| 6951195 | 30.0 | 18.0 | 5.8 | 12.0 | 14.0 |
| 7147179 | 0.6 | 0.0 | 0.0 | 0.0 | 1.0 |
| 7147180 | 0.6 | 0.0 | 1.2 | 0.0 | 1.0 |
| 7147182 | 1.2 | 0.0 | 0.0 | 0.0 | 1.3 |
