# Supplementary material for: Multiple-Race Stem Rust Resistance Loci Identified in Durum Wheat Using Genome-Wide Association Mapping
Source: Front Plant Sci. 2020 Dec 17;11:598509. doi: 10.3389/fpls.2020.598509 (PMC7773921; doi:10.3389/fpls.2020.598509)
Supplement: Supplementary Table 2 — Lists of SNPs significantly associated with field resistance to East African Pgt races across five seasons identified using FarmCPU. [file Table_2.docx]

| Supplementary TABLE 2. Lists of SNPs significantly associated with field resistance to East African *Pgt* races across five seasons identified using FarmCPU. | | | | | | | |
| --- | --- | --- | --- | --- | --- | --- | --- |
| Env. | Position (bp) | Chr. | P value | Allele | AF | Effect | Proposed gene |
| ETOS18 | 20977834 | 2A | 1.17E-07 | **C**/T | 0.721 | 2.16 | Letta et al. (2014) |
|  | 135744411 | 2A | 2.12E-08 | G/**A** | 0.355 | -2.72 | Likely novel |
|  | 724805496 | 3B | 5.57E-09 | G/**A** | 0.104 | -4.35 | *Sr12* |
|  | 13909625 | 5B | 5.43E-06 | **T/**C | 0.751 | 2.36 | Yu et al. (2014) |
|  | 691693264 | 5B | 1.92E-08 | T/**A** | 0.051 | -6.24 | *Sr49* |
|  | 592006 | 6A | 4.28E-07 | G/**A** | 0.228 | -2.43 | Novel*/Sr8155B1* |
|  | 1424376 | 6A | 3.47E-06 | **C**/G | 0.906 | 3.79 | *Sr8a* |
|  | 610171399 | 6A | 5.10E-13 | **G**/A | 0.820 | 4.53 | *Sr13* |
|  | 613576841 | 6A | 4.27E-16 | **G**/C | 0.813 | 4.98 | *Sr13* |
|  | 31294519 | 6B | 7.81E-06 | **C**/T | 0.790 | 2.01 | Likely novel |
|  | 700805183 | 7A | 1.34E-31 | A/**T** | 0.076 | -12.19 | *Sr22* |
| ETMS18 | 95587608 | 1A | 6.82E-06 | **A**/G | 0.937 | 0.38 | Likely novel |
|  | 144772265 | 1A | 1.63E-05 | **A**/G | 0.931 | 0.37 | - |
|  | 313477146 | 3A | 1.90E-06 | **C**/T | 0.841 | 0.21 | Likely novel |
|  | 344594454 | 3A | 1.02E-06 | T/**G** | 0.108 | -0.33 | Likely novel |
|  | 691693264 | 5B | 1.90E-05 | T/**A** | 0.051 | -0.37 | *Sr49* |
|  | 598562950 | 6A | 6.46E-07 | **A**/G | 0.544 | 0.16 | Likely novel |
|  | 609346836 | 6A | 4.04E-06 | **C**/G | 0.894 | 0.31 | *Sr13 allele* |
|  | 612043936 | 6A | 8.05E-20 | T/**C** | 0.302 | -0.48 | *Sr13* |
|  | 615604035 | 6A | 1.02E-06 | A/**C** | 0.274 | -0.20 | *Sr13* |
|  | 30564627 | 6B | 1.15E-09 | **A**/G | 0.562 | 0.23 | Likely novel |
|  | 717518884 | 7A | 1.08E-15 | T/**C** | 0.058 | -0.83 | *Sr22* |
|  | 717849029 | 7B | 1.89E-06 | T/**G** | 0.081 | -0.32 | Bajgain et al. (2015b) |
| ETOS19 | 78492640 | 2A | 9.73E-08 | **A**/C | 0.940 | 0.47 | Likely novel |
|  | 456530846 | 2B | 9.29E-06 | **A**/G | 0.913 | 0.35 | - |
|  | 35001659 | 5A | 1.65E-05 | **T**/G | 0.820 | 0.24 | Likely novel |
|  | 692277095 | 5B | 3.36E-07 | T/**C** | 0.058 | -0.40 | *Sr49* |
|  | 606107662 | 6A | 2.21E-10 | **G**/A | 0.636 | 0.33 | *Sr13* |
|  | 611495915 | 6A | 1.29E-17 | **G**/A | 0.846 | 0.70 | *Sr13* |
|  | 612003938 | 6A | 4.42E-10 | G/**A** | 0.095 | -0.50 | *Sr13 allele* |
|  | 612802438 | 6A | 3.25E-33 | **A**/C | 0.708 | 0.80 | Novel/*Sr13b* |
|  | 692192009 | 6B | 1.10E-09 | A/**G** | 0.053 | -0.56 | *Sr11* |
|  | 673523659 | 7A | 1.51E-08 | T/**A** | 0.092 | -0.44 | Likely *Sr22* |
|  | 700805183 | 7A | 5.95E-17 | A/**T** | 0.076 | -0.87 | *Sr22* |
|  | 46338417 | 7B | 5.51E-07 | C/**T** | 0.417 | -0.21 | Likely novel |
| KNMS18 | 9819941 | 3A | 6.15E-07 | **A**/G | 0.846 | 0.39 | Letta et al. (2013) |
|  | 724805496 | 3B | 5.46E-06 | G/**A** | 0.104 | -0.43 | *Sr12* |
|  | 8470400 | 5A | 4.72E-08 | T/**C** | 0.416 | -0.29 | Likely novel |
|  | 12999566 | 5B | 2.55E-06 | **C**/T | 0.878 | 0.44 | Yu et al. (2014) |
|  | 692277095 | 5B | 3.47E-07 | T/**C** | 0.058 | -0.61 | *Sr49* |
|  | 4914394 | 6A | 4.22E-11 | C/**G** | 0.226 | -0.45 | *Sr8155B1* |
|  | 609622362 | 6A | 9.13E-06 | **T**/C | 0.829 | 0.33 | *Sr13 allele* |
|  | 612043936 | 6A | 1.25E-10 | T/**C** | 0.302 | -0.44 | *Sr13* |
|  | 615619215 | 6A | 1.61E-05 | **G**/A | 0.820 | 0.31 | *Sr13* |
|  | 666439193 | 6B | 2.17E-06 | G/**A** | 0.378 | -0.26 | Likely novel |
|  | 717518884 | 7A | 2.26E-26 | T/**C** | 0.058 | -1.48 | *Sr22* |
| KNMS19 | 546977269 | 1B | 2.92E-08 | **C**/T | 0.869 | 0.47 | Letta et al. (2014) |
|  | 770363872 | 2A | 1.60E-07 | C/**G** | 0.071 | -0.67 | Likely novel |
|  | 759454292 | 2B | 5.78E-11 | **A**/G | 0.756 | 0.43 | *SrWeb/Sr9h* |
|  | 581703945 | 5B | 1.48E-05 | **G**/A | 0.913 | 0.49 | Reported APR |
|  | 671134916 | 5B | 7.69E-06 | **C**/G | 0.611 | 0.23 | Likely novel |
|  | 592006 | 6A | 8.96E-09 | G/**A** | 0.228 | -0.36 | Novel*/Sr8155B1* |
|  | 612043936 | 6A | 1.06E-13 | T/**C** | 0.302 | -0.49 | *Sr13* |
|  | 285980279 | 7A | 4.64E-06 | **A**/T | 0.882 | 0.40 | Likely novel |
|  | 700805183 | 7A | 2.22E-17 | A/**T** | 0.076 | -1.08 | *Sr22* |
|  | 122277080 | UN | 5.55E-06 | **G**/T | 0.936 | 0.53 | Unknown |

AF= allele frequency, bold face written alleles are the favorable allele at each locus.
