# Supplementary material for: Multiple-Race Stem Rust Resistance Loci Identified in Durum Wheat Using Genome-Wide Association Mapping
Source: Front Plant Sci. 2020 Dec 17;11:598509. doi: 10.3389/fpls.2020.598509 (PMC7773921; doi:10.3389/fpls.2020.598509)
Supplement: Supplementary Table 8 — Lists of consistent significant markers between testing environments identified using MLM. [file Table_8.docx]

| Supplementary TABLE 8. Lists of consistent significant markers between testing environments identified using MLM. | | | | |
| --- | --- | --- | --- | --- |
| Position | Chr. | MAF | Environment | Proposed gene |
| 97870708 | 3B | 0.055 | ETOS18, ETMS18, ETOS19, KNMS18 | Novel |
| 619746683 | 4A | 0.053 | ETOS18, ETMS18, ETOS19, KNMS18, KNMS19 | Novel |
| 28859024 | 6A | 0.051 | ETOS18, KNMS18 | Novel |
| 334834338 | 6A | 0.051 | ETOS18, ETMS18, ETOS19, KNMS18, KNMS19 | Novel |
| 609622362 | 6A | 0.171 | ETOS18, ETOS19 | *Sr13* |
| 609635640 | 6A | 0.154 | ETOS18, ETMS18, ETOS19 |  |
| 610133407 | 6A | 0.166 | ETMS18, ETOS19 |  |
| 610171399 | 6A | 0.180 | ETOS18, ETOS19 |  |
| 610495870 | 6A | 0.177 | ETOS18, ETOS19 |  |
| 611495915 | 6A | 0.154 | ETMS18, ETOS19 |  |
| 612043936 | 6A | 0.302 | ETOS18, ETMS18, KNMS18, KNMS19 |  |
| 612802438 | 6A | 0.292 | ETOS18, ETOS19, KNMS18 |  |
| 612832613 | 6A | 0.261 | ETOS18, ETOS19 |  |
| 612957317 | 6A | 0.265 | ETOS18, ETOS19 |  |
| 613055519 | 6A | 0.263 | ETOS18, ETOS19 |  |
| 613131839 | 6A | 0.261 | ETOS18, ETOS19 |  |
| 613194512 | 6A | 0.261 | ETOS18, ETOS19 |  |
| 613256520 | 6A | 0.274 | ETOS18, ETOS19, KNMS18 |  |
| 613288180 | 6A | 0.173 | ETOS18, ETOS19 |  |
| 613294106 | 6A | 0.169 | ETOS18, ETOS19 |  |
| 613294155 | 6A | 0.265 | ETOS18, ETOS19 |  |
| 613547583 | 6A | 0.175 | ETOS18, ETOS19 |  |
| 613576841 | 6A | 0.187 | ETOS18, ETOS19 |  |
| 614329660 | 6A | 0.203 | ETOS18, ETOS19 |  |
| 615604386 | 6A | 0.311 | ETOS18, ETOS19 |  |
| 615617605 | 6A | 0.184 | ETOS18, ETOS19 |  |
| 615619215 | 6A | 0.180 | ETOS18, ETOS19 |  |
| 690016567 | 7A | 0.051 | ETOS18, KNMS18, KNMS19 | *Sr22* |
| 690811708 | 7A | 0.060 | ETOS18, ETMS18, KNMS18, KNMS19 |  |
| 690940195 | 7A | 0.057 | ETOS18, ETMS18, ETOS19, KNMS18, KNMS19 |  |
| 693915965 | 7A | 0.071 | ETOS18, ETMS18 |  |
| 697030510 | 7A | 0.092 | ETOS18, ETMS18, ETOS19, KNMS18 |  |
| 697030516 | 7A | 0.054 | ETOS18, ETMS18, ETOS19, KNMS18, KNMS19 |  |
| 698390754 | 7A | 0.095 | ETOS18, ETMS18, ETOS19, KNMS18 |  |
| 700727874 | 7A | 0.058 | ETOS18, ETMS18, ETOS19, KNMS18, KNMS19 |  |
| 700805183 | 7A | 0.076 | ETOS18, ETMS18, ETOS19, KNMS18, KNMS19 |  |
| 710171609 | 7A | 0.055 | ETOS18, ETMS18, ETOS19, KNMS18, KNMS19 |  |
| 714327927 | 7A | 0.065 | ETOS18, ETMS18, ETOS19, KNMS18, KNMS19 |  |
| 714370100 | 7A | 0.051 | ETOS18, ETMS18, ETOS19, KNMS18, KNMS19 |  |
| 714975616 | 7A | 0.901 | ETOS18, ETMS18, ETOS19, KNMS18 |  |
| 717517491 | 7A | 0.054 | ETOS18, ETMS18, ETOS19, KNMS18, KNMS19 |  |
| 717518884 | 7A | 0.058 | ETOS18, ETMS18, ETOS19, KNMS18, KNMS19 |  |
| 718484217 | 7A | 0.097 | ETOS18, ETMS18, ETOS19, KNMS19 |  |
| 719231181 | 7A | 0.067 | ETOS18, ETMS18, ETOS19, KNMS18 |  |
| 719698163 | 7A | 0.058 | ETOS18, ETMS18, ETOS19, KNMS18, KNMS19 |  |
| 719787589 | 7A | 0.051 | ETOS18, ETMS18, KNMS18, KNMS19 |  |
| 721720978 | 7A | 0.064 | ETOS18, ETMS18, ETOS19, KNMS18, KNMS19 |  |
| 724486791 | 7A | 0.104 | ETOS18, ETMS18 |  |
| 724668618 | 7A | 0.059 | ETOS18, ETMS18, ETOS19, KNMS18, KNMS19 |  |
| 724668652 | 7A | 0.051 | ETOS18, ETMS18, ETOS19, KNMS18, KNMS19 |  |
| 622041448 | 7B | 0.074 | ETOS18, ETMS18, KNMS18 | *Sr17* |
| 644041948 | 7B | 0.058 | ETOS18, ETMS18, KNMS19, KNMS19 |  |
| 151742792 | UN | 0.054 | ETOS18, ETMS18, ETOS19, KNMS18, KNMS19 | Unknown |
| 151847140 | UN | 0.056 | ETOS18, ETMS18, ETOS19, KNMS18, KNMS19 |  |
| 153093563 | UN | 0.099 | ETOS18, ETMS18, KNMS19 |  |
| 153928527 | UN | 0.054 | ETOS18, ETMS18, ETOS19, KNMS18, KNMS19 |  |
| 166522707 | UN | 0.053 | ETOS18, ETMS18, ETOS19, KNMS18, KNMS19 |  |
